# The Role of HINT3 in Myocardial Ischemia‐Reperfusion Injury in Male Mice: Mechanisms Involving SDHA and its Acetylation

**DOI:** 10.1002/advs.202503109

**Published:** 2025-08-04

**Authors:** Jiabin Yu, Qi Yao, Tongtong Hu, Yadan Zhang, Ying Liu, Yang Xiao, Qingqing Wu, Qizhu Tang

**Affiliations:** ^1^ Department of Cardiology Renmin Hospital of Wuhan University Wuhan 430060 P. R. China; ^2^ Hubei Key Laboratory of Metabolic and Chronic Diseases Wuhan 430060 P. R. China; ^3^ Taikang Center for Life and Medical Sciences Taikang Medical School Wuhan University Wuhan 430072 P. R. China

**Keywords:** acetylation, HINT3, mitochondrial dysfunction, myocardial ischemia/reperfusion, SDHA

## Abstract

Myocardial ischemia‐reperfusion (I/R) injury is characterized by oxidative stress, mitochondrial dysfunction, and cardiomyocyte apoptosis. During I/R, the accumulation and oxidation of succinate contribute to reactive oxygen species (ROS) production, worsening tissue damage. Histidine triad nucleotide‐binding protein 3 (HINT3) is identified as a regulator of mitochondrial function and cardiomyocyte survival during I/R. In a mouse I/R model and an oxygen‐glucose deprivation/reoxygenation (OGD/R) model, it shows that HINT3 expression is downregulated after I/R. Cardiomyocyte‐specific knockout of HINT3 exacerbates myocardial injury, impairs cardiac function, and promotes mitochondrial dysfunction and apoptosis, whereas HINT3 overexpression mitigates these effects. Mechanistically, HINT3 interacts with succinate dehydrogenase subunit A (SDHA), suppresses HDAC1 expression, and prevents SDHA deacetylation at K335, reducing SDH activity and mitochondrial ROS production. These findings highlight the HINT3‐HDAC1‐SDHA axis as a key pathway in mitochondrial regulation, offering new insights and therapeutic targets for myocardial reperfusion injury.

## Graphical abstract

HINT3 expression declines after myocardial ischemia‐reperfusion injury. In male mice, loss of HINT3 worsens cardiac injury and mitochondrial dysfunction, while its overexpression is protective. HINT3 binds the mitochondrial enzyme succinate dehydrogenase (SDHA) and prevents its deacetylation by histone deacetylase 1 (HDAC1), reducing succinate dehydrogenase activity and reactive oxygen species generation. These findings reveal a protective HINT3–HDAC1–SDHA axis in mitochondrial regulation.

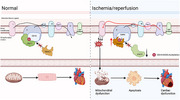

## 1 Introduction

The primary goal of myocardial infarction treatment is to restore cardiac blood flow as quickly as possible to minimize the extent of myocardial damage. Despite these inherent benefits, timely reperfusion can paradoxically cause additional myocardial damage, a phenomenon known as myocardial ischemia‐reperfusion injury (MIRI).^[^ ^1^ ^]^ The pathological and molecular mechanism of MIRI has become increasingly elucidated, including mechanisms such as oxidative stress,^[^ ^2^ ^]^ inflammation,^[^ ^3^ ^]^ calcium overload,^[^ ^4^ ^]^ endothelial dysfunction,^[^ ^5^ ^]^ and impaired microvascular blood flow.^[^ ^6^ ^]^ Although numerous pathophysiological factors are involved, mitochondrial reactive oxygen species (ROS) production has been identified as the initial trigger for a cascade of events leading to tissue damage during I/R injury.^[^ ^7^ ^]^ The initial burst of ROS production during reperfusion directly induces oxidative damage to mitochondria, thereby disrupting ATP generation.^[^ ^8^ ^]^ Together with calcium dysregulation, elevated ROS can trigger mitochondrial permeability transition (MPT).^[^ ^9^ ^]^ Collectively, these mitochondrial dysfunction events lead to cardiomyocyte necrosis and apoptosis upon reperfusion.

Histidine triad nucleotide‐binding proteins (HINT) belong to the histidine triad (HIT) superfamily, characterized by a conserved C‐terminal active site motif His‐X‐His‐X‐His‐XX, where X represents hydrophobic residues. The HIT superfamily primarily consists of nucleoside phosphoramidases, dinucleoside hydrolases, and nucleotide transferases.^[^ ^10^ ^]^ Members of the HINT family are widely involved in the occurrence and development of tumors, playing crucial roles in tumor suppression. For instance, Li et al. demonstrated that HINT3 inhibits the activation of the PTEN/AKT/mTOR signaling pathway and suppresses the proliferation, growth, migration, and tumorigenicity of MCF‐7 and MDA‐MB‐231 breast cancer cells.^[^ ^11^ ^]^ Similarly, Jung et al. found that the deacetylation of SIRT1 enhances the tumor suppressor activity of HINT1 by promoting its interaction with *β*‐catenin or MITF in colon cancer and melanoma cells.^[^ ^12^ ^]^ Beyond their roles in tumor‐related diseases, the protective effects of HINT family members in cardiovascular diseases have gained increasing attention in recent years. For example, HINT1 has been shown to prevent cardiac hypertrophy by inhibiting HOXA5 expression.^[^ ^13^ ^]^ HINT2 overexpression mitigates the progression of cardiac microvascular ischemia‐reperfusion injury by suppressing the MCU complex‐mediated mitochondrial calcium overload, mitochondrial fission, and apoptosis pathway. Additionally, in a mouse model of pressure overload‐induced cardiac hypertrophy, we observed that HINT2 alleviates cardiac remodeling by modulating the activity and assembly of mitochondrial complex I.^[^ ^14^ ^]^ In contrast to HINT1 and HINT2, research on HINT3 is very limited, especially concerning its role in cardiovascular disease – a role that remains virtually unexplored.

In this study, we utilized a mouse model of myocardial ischemia‐reperfusion injury and a cellular model of oxygen‐glucose deprivation/reoxygenation (OGD/R) to investigate the role of HINT3 in reperfusion injury. Our findings reveal that cardiomyocyte‐specific deletion of HINT3 exacerbates myocardial reperfusion injury, increases apoptosis, and causes mitochondrial dysfunction. In contrast, overexpression of HINT3 alleviates myocardial reperfusion injury. Using immunoprecipitation‐mass spectrometry (IP‐MS) and the STRING database, we identified the mitochondrial protein SDHA as a key interaction partner of HINT3. Recent studies have revealed that the mitochondrial metabolite succinate accumulates significantly in tissues during ischemia and is rapidly oxidized by succinate dehydrogenase (SDH) upon reperfusion. This oxidation drives superoxide production at mitochondrial complex I via reverse electron transport (RET), thereby triggering ischemia‐reperfusion injury.^[^ ^15^, ^16^, ^17^ ^]^ Growing evidence suggests that inhibiting succinate metabolism during reperfusion using malonate can effectively reduce mitochondrial reactive oxygen species (ROS) production.^[^ ^18^, ^19^, ^20^, ^21^ ^]^ Therefore, targeting the production and metabolism of succinate may hold significant therapeutic potential for addressing myocardial ischemia‐reperfusion injury. Furthermore, we found that HINT3 suppresses HDAC1 protein expression and competitively interacts with SDHA, thereby inhibiting HDAC1‐mediated deacetylation of SDHA at Lys335. This regulation reduces SDH activity and mitigates mitochondrial ROS production, ultimately protecting against myocardial ischemia‐reperfusion injury.

## 2 Results

### 2.1 HINT3 Expression is Downregulated in Cardiac Tissue and Cardiomyocytes Following Ischemia‐Reperfusion Injury

**Figure 1:**
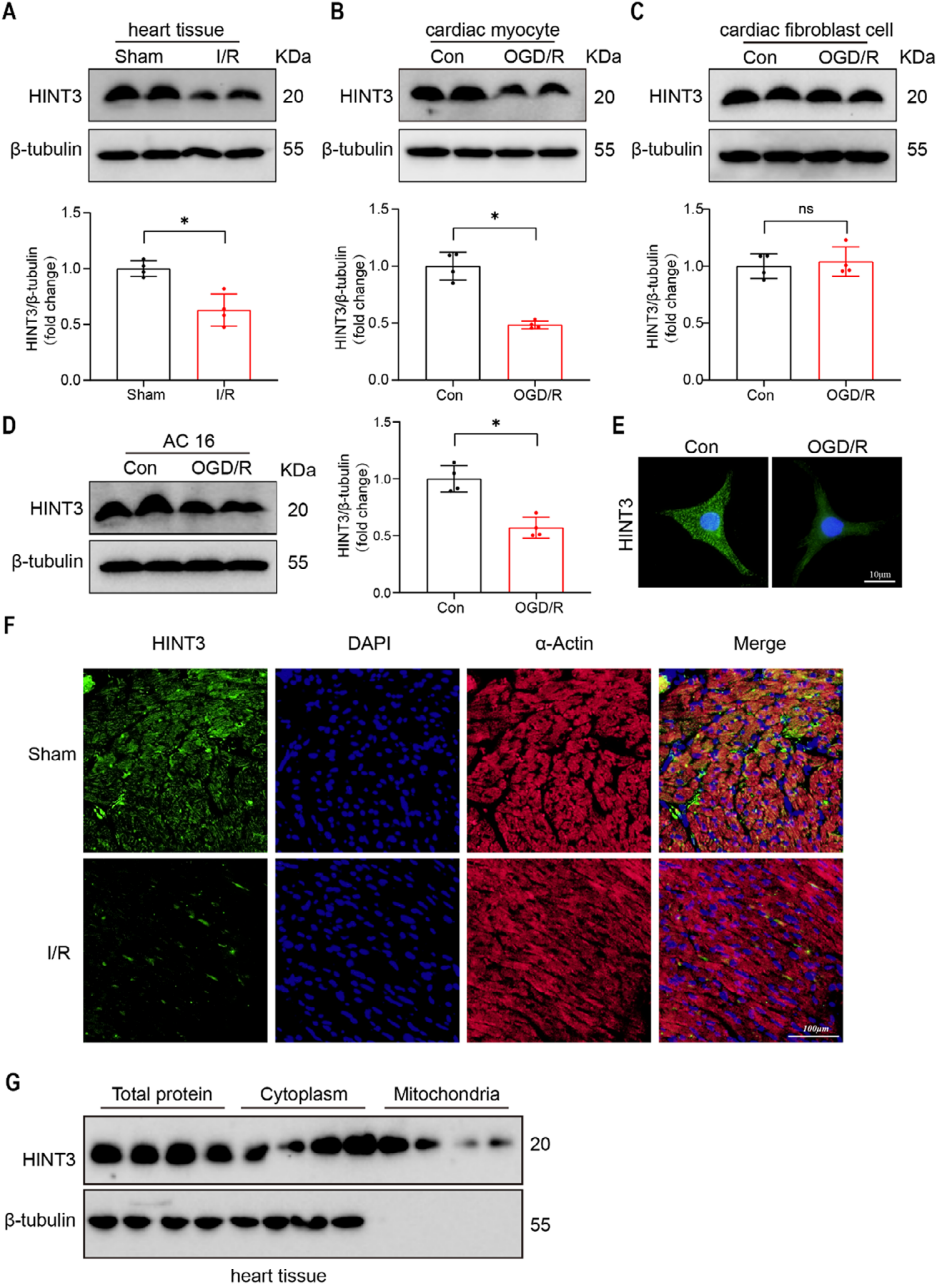
HINT3 expression is downregulated in cardiac tissue and cardiomyocytes under I/R conditions. A) Representative Western blot and quantification showing a significant decrease in HINT3 protein expression in heart tissue of C57BL/6 mice subjected to I/R surgery compared to sham group (n = 4). B) Western blot analysis of HINT3 protein levels in neonatal rat cardiomyocytes (NRCMs) exposed to OGD/R or control conditions (n = 4). C) HINT3 expression in neonatal rat cardiac fibroblasts subjected to control or OGD/R treatment (n = 4). D) Western blot and quantification of HINT3 expression in human cardiomyocyte cell line AC16 under control and OGD/R conditions (n = 4). E) Representative immunofluorescence staining showing reduced HINT3 (green) fluorescence in AC16 cells following OGD/R. Nuclei stained with DAPI (blue). Scale bar: 10 µm. F) Immunofluorescence staining of cardiac tissue sections from sham and I/R mice showing decreased HINT3 (green) expression in α‐actinin⁺ cardiomyocytes (red). Nuclei stained with DAPI (blue). Scale bar: 100 µm. G) Western blot analysis of HINT3 subcellular distribution in mouse heart tissue. (*P < 0.05).

To explore the potential involvement of HINT family members in myocardial I/R injury, we first analyzed transcriptomic data from the GEO database (GSE255933). Among the HIT proteins, HINT3 exhibited notable downregulation in mouse heart tissues after I/R injury (Figure S1A, Supporting Information). Given the limited prior research on HINT3 in the cardiovascular system, we selected HINT3 for further investigation. We then evaluated HINT3 expression in mouse cardiac tissue following ischemia‐reperfusion injury. HINT3 expression was significantly reduced in heart tissue from mice subjected to I/R injury compared to the control group (**Figure** **1**A). To confirm these findings, we isolated primary cardiomyocytes and fibroblasts from neonatal rat hearts and subjected them to an OGD/R model of I/R injury. In primary cardiomyocytes, HINT3 expression was markedly decreased following OGD/R treatment (Figure 1B), whereas no detectable change was observed in fibroblasts (Figure 1C), indicating that the downregulation of HINT3 is specific to cardiomyocytes under I/R conditions. We further validated these results in a human cardiomyocyte cell line (AC16), where HINT3 protein levels were also significantly decreased following OGD/R (Figure 1D). Immunofluorescence analysis also demonstrated reduced HINT3 fluorescence intensity under OGD/R conditions, consistent with the protein expression data (Figure 1E). Also, immunofluorescence staining of cardiac tissue sections from mice subjected to I/R injury revealed a marked reduction in HINT3 expression, corroborating our in vitro findings (Figure 1F). To determine the subcellular localization of HINT3, we performed subcellular fractionation of mouse heart tissue and found that HINT3 is primarily localized in the cytoplasm, with a small fraction detected in mitochondria (Figure 1G). Additionally, to evaluate cell‐type‐specific expression of HINT3, we performed immunofluorescence staining of heart sections using markers for endothelial cells (CD31), fibroblasts (Vimentin). We found that HINT3 is minimally expressed in CD31⁺ and showed no significant change in Vimentin⁺ fibroblasts under I/R conditions (Figure S1B,C, Supporting Information). Finally, tissue‐wide profiling of HINT3 in mouse organs demonstrated that HINT3 is broadly expressed, with heart tissue exhibiting moderate levels (Figure S1D, Supporting Information). Collectively, these results indicate that HINT3 expression is downregulated in mouse cardiac tissue and cardiomyocytes in response to ischemia‐reperfusion injury.

### 2.2 Cardiomyocyte‐Specific HINT3 Knockout Exacerbates Myocardial Damage and Mitochondrial Dysfunction After I/R Injury

**Figure 2:**
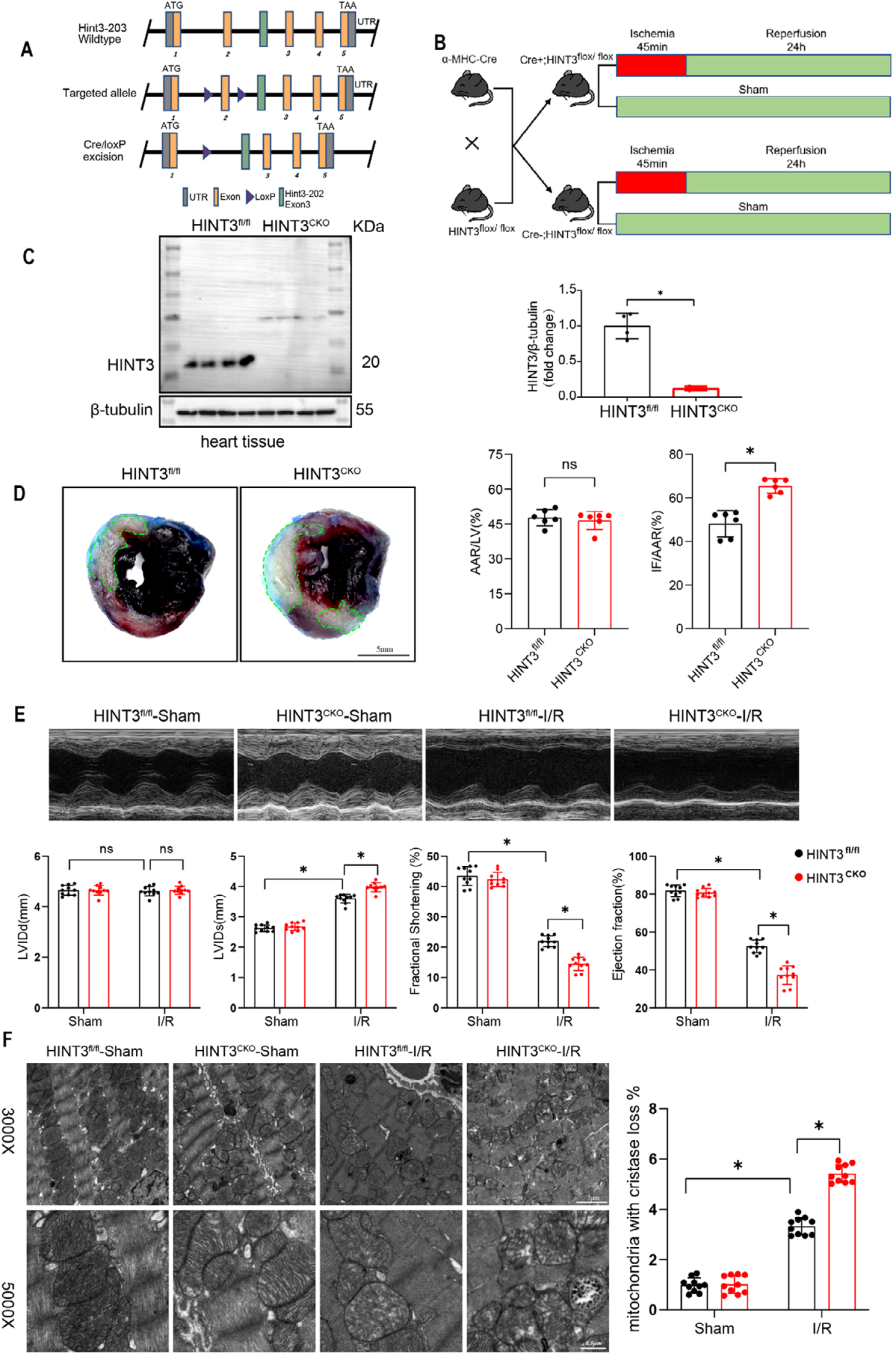
Cardiomyocyte‐specific knockout of HINT3 exacerbates myocardial I/R injury. A) Schematic diagram illustrating the generation of HINT3^fl/fl^ mice and Cre‐loxP‐mediated HINT3 knockout (HINT3^CKO^) in cardiomyocytes. B) Diagram showing the experimental protocol for myocardial I/R injury (45 min ischemia followed by 24 h reperfusion). C) Western blot analysis of HINT3 expression in hearts from HINT3 cardiomyocyte‐specific knockout (HINT3^CKO^) mice and their HINT3^fl/fl^ littermate controls (n = 4). D) Representative TTC/EB‐stained heart sections showing infarct areas (white), which are outlined by green dashed lines, and quantification of infarct size (IF)/area at risk (AAR) and AAR/left ventricle (LV) ratios in HINT3^fl/fl^ and HINT3^CKO^ mice 24 h after I/R injury (n = 6). E) Representative M‐mode echocardiography images and quantification of cardiac function, including left ventricular end‐diastolic diameter (LVIDd), left ventricular end‐systolic diameter (LVIDs), fractional shortening (FS), and ejection fraction (EF), in HINT3^CKO^ and HINT3^fl/fl^ mice under sham and I/R conditions (n = 10). F) Transmission electron microscopy (TEM) images showing mitochondrial ultrastructure at 3000× and 5000× magnification, and quantification of mitochondria with cristae loss in mouse hearts (n =10). Scale bars: 2 µm (3000×) and 0.5 µm (5000×).

To investigate the role of HINT3 in myocardial I/R injury, we generated a cardiomyocyte‐specific HINT3 knockout mouse model (HINT3^CKO^) by breeding HINT3^fl/fl^ mice with cardiomyocyte‐specific Cre recombinase mice. Mice were treated with tamoxifen (20 mg/kg/d) via daily intraperitoneal injection for five consecutive days to induce the knockout (Figure **2**A,C; Figure S1E, Supporting Information). We next subjected HINT3^CKO^ mice and control littermates to cardiac I/R injury (Figure 2B). Then, we assessed the infarction area in I/R injured hearts using TTC‐EB staining. TTC‐EB stain showed that HINT3^CKO^ mice exhibited a significantly larger infarct area following reperfusion, as indicated by the infarct size (IF) to area at risk (AAR) ratio. The AAR to left ventricular (LV) ratio did not differ significantly between the groups, suggesting that the increase in infarct size was specific to the myocardial damage induced by I/R (Figure 2D). Cardiac function was further evaluated by echocardiography. HINT3^CKO^ mice exhibited significantly worsened cardiac function compared to control mice, as evidenced by a significant increase in left ventricular internal diameter at systole (LVIDs) and a notable decrease in both fractional shortening (FS) and ejection fraction (EF) (Figure 2E). To further evaluate the extent of myocardial injury, we measured the levels of cardiac damage biomarkers in mouse serum. As shown in (Figure S2A–C, Supporting Information), I/R significantly increased serum levels of lactate dehydrogenase (LDH), creatine kinase‐MB (CK‐MB), and cardiac troponin T (cTnT) compared to the sham group. Notably, these elevations were more pronounced in HINT3^CKO^ mice compared to HINT3^fl/fl^ controls. Finally, mitochondrial morphology in the hearts of HINT3^CKO^ mice was assessed using transmission electron microscopy (TEM), which revealed more disorganized mitochondrial structures and an increased loss of mitochondrial cristae, further indicating that the absence of HINT3 exacerbates myocardial injury at the cellular level (Figure 2F).

### 2.3 HINT3 Knockout Promotes Apoptosis and Mitochondrial Dysfunction Through Transcriptomic and Functional Analyses

**Figure 3:**
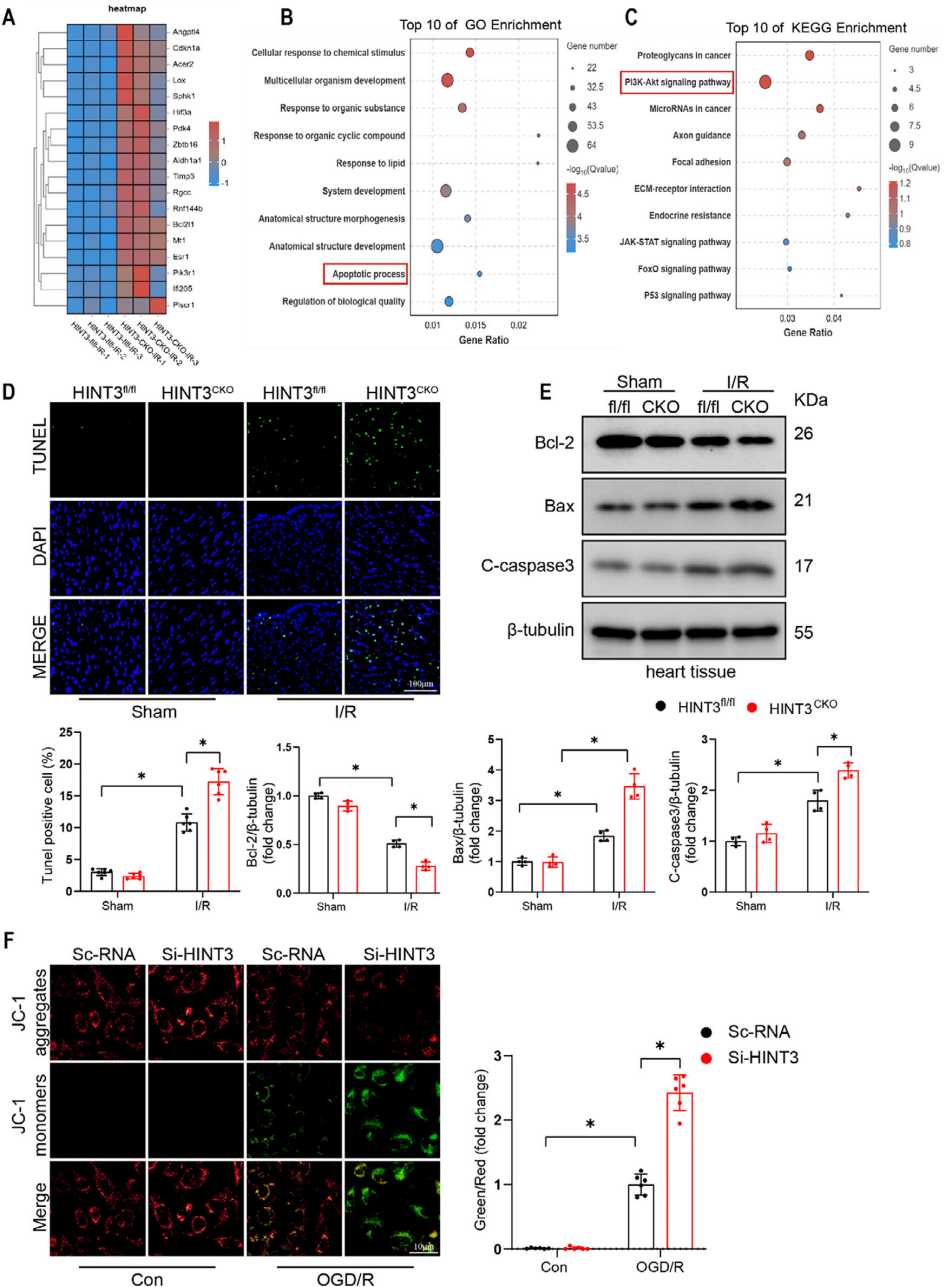
HINT3 knockout promotes apoptosis and mitochondrial dysfunction in response to I/R injury. A) Heatmap showing the expression levels of differentially expressed genes (DEGs) in cardiac tissue from HINT3^fl/fl^ and HINT3^CKO^ mice under I/R conditions. Each row represents a gene, and each column represents a sample, with red indicating upregulation and blue indicating downregulation of gene expression. B) Gene Ontology (GO) enrichment analysis of DEGs, highlighting biological processes such as response to chemical stimulus, multicellular organism development, response to organic substances, and regulation of biological quality. Dot size represents the number of genes, and the color gradient indicates statistical significance (‐log10 Q‐value). C) KEGG pathway enrichment analysis of DEGs, showing pathways such as PI3K‐Akt signaling, proteoglycans in cancer, focal adhesion, and JAK‐STAT signaling. Dot size corresponds to the number of genes in each pathway, and the color gradient reflects statistical significance (‐log10 Q‐value). D) TUNEL staining showing apoptotic cardiomyocytes in HINT3^fl/fl^ and HINT3^CKO^ hearts post‐I/R, with quantification of TUNEL‐positive cells (n = 6). Scale bar: 100 µm. E) Representative Western blot and quantitative analysis of apoptosis‐related proteins, including Bcl‐2, Bax, and cleaved caspase‐3 (C‐caspase3), in mouse hearts (n = 4). F) JC‐1 staining in AC16 cells transfected with si‐HINT3 or Sc‐RNA under OGD/R, showing impaired mitochondrial membrane potential indicated by increased green/red fluorescence ratio (n = 6), data are presented as fold change relative to OGD/R + Sc‐RNA group.

To explore the involvement of HINT3 in regulating I/R injury, we performed RNA sequencing (RNA‐seq) on cardiac tissue from HINT3^fl/fl^ and HINT3^CKO^ mice following I/R injury. Our analysis revealed that the cardiomyocyte‐specific knockout of HINT3 led to the upregulation of 115 genes and the downregulation of 21 genes (Figure S1G, Supporting Information). Gene Ontology (GO) and Kyoto Encyclopedia of Genes and Genomes (KEGG) pathway enrichment analyses highlighted that apoptosis‐related pathways were significantly enriched in the HINT3^CKO^ group after I/R injury. Apoptosis‐related pathways, such as those regulated by mitochondrial dysfunction, have been widely implicated in I/R‐induced injury.^[^ ^22^, ^23^ ^]^ Heatmap analysis of apoptosis‐related genes further confirmed a marked upregulation of these genes in HINT3^CKO^ mice after I/R (Figure **3**A‐C). Histopathological analysis of cardiac tissue using TUNEL staining revealed a significant increase in apoptotic cells in the hearts of HINT3^CKO^ mice following I/R injury (Figure 3D), supporting the RNA‐seq findings. Next, we examined the expression of apoptosis‐related proteins in response to HINT3 knockout. The absence of HINT3 exacerbated I/R‐induced apoptosis, as evidenced by a significant downregulation of the anti‐apoptotic protein BCL‐2 and a marked increase in the pro‐apoptotic proteins BAX and cleaved caspase‐3 (Figure 3E). We observed similar changes in our cellular experiments (Figure S2E‐G, Supporting Information). This is consistent with the established role of BCL‐2 and BAX in regulating mitochondria‐mediated apoptosis during oxidative stress.^[^ ^24^ ^]^ To assess the impact of HINT3 deletion on oxidative stress in myocardial tissue, dihydroethidium (DHE) staining was performed on heart sections from HINT3^fl/fl^ and HINT3^CKO^ mice. I/R injury markedly increased reactive oxygen species (ROS) levels in both groups. However, HINT3^CKO^ hearts exhibited a substantially stronger red fluorescence signal compared to controls, indicating exacerbated ROS accumulation in the absence of HINT3 (Figure S2D, Supporting Information). To further investigate the effect of HINT3 on mitochondrial function, we knocked down HINT3 using siRNA in AC16 cells and subjected them to OGD/R, followed by an assessment of mitochondrial membrane potential (MMP) using JC‐1 staining. The reduction in mitochondrial membrane potential induced by OGD/R was further exacerbated by HINT3 knockdown, as indicated by the Green/Red fluorescence ratio, suggesting a detrimental effect on mitochondrial function (Figure 3F).

### 2.4 HINT3 Overexpression Mitigates Myocardial Damage and Preserves Mitochondrial Integrity During I/R Injury

**Figure 4:**
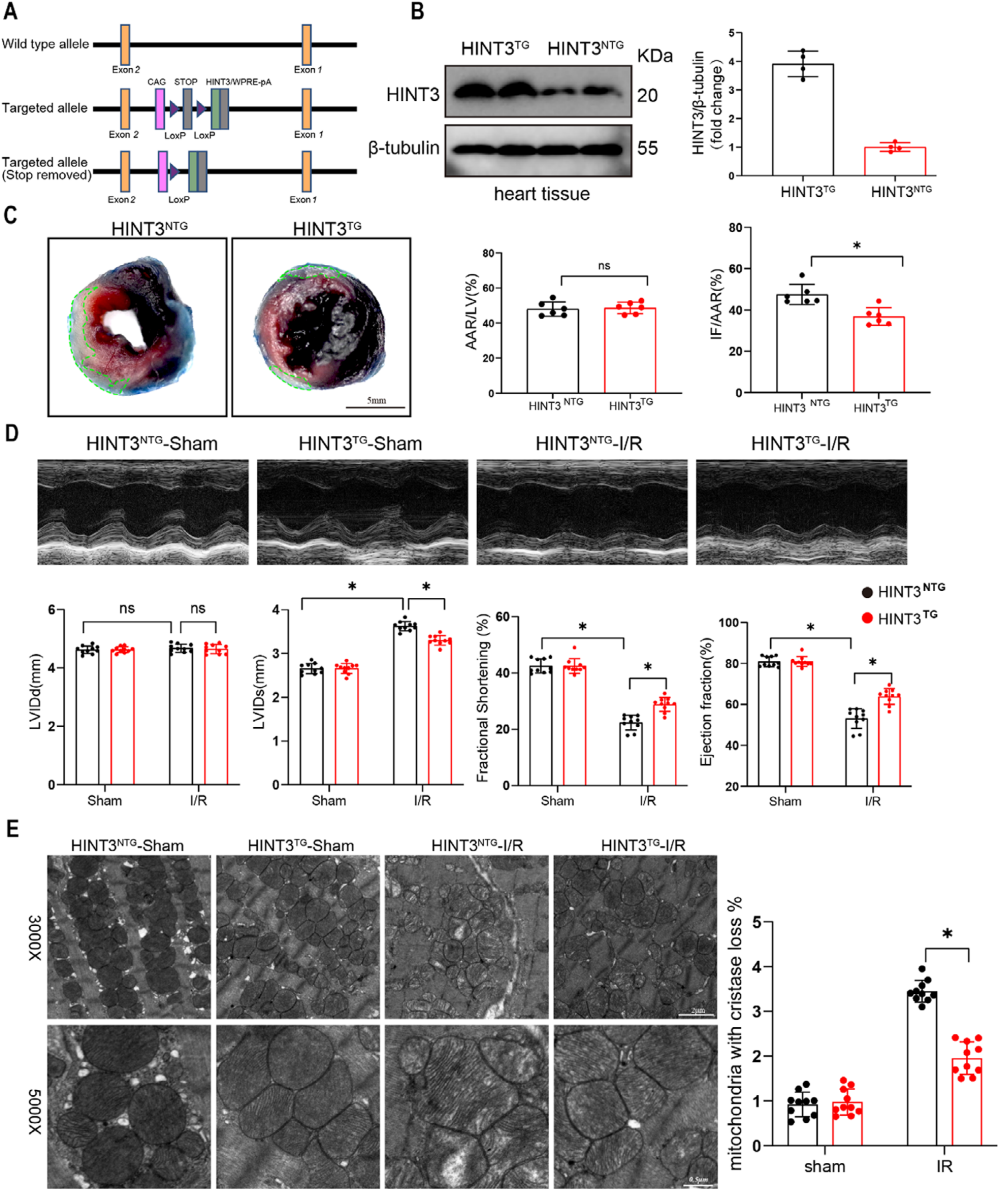
Cardiomyocyte‐specific overexpression of HINT3 alleviates myocardial I/R injury. A) Schematic representation illustrating the construction of the transgenic mouse model overexpressing HINT3 in cardiomyocytes. B) Representative Western blot analysis showing HINT3 expression in hearts of HINT3^TG^ and HINT3^NTG^ mice (n = 4). C) Representative TTC/EB‐stained heart sections showing infarct areas (white), which are outlined by green dashed lines, and quantification of infarct size (IF)/area at risk (AAR) and AAR/left ventricle (LV) ratios in HINT3^NTG^ and HINT3^TG^ mice 24 h after I/R injury (n = 6).D) Representative M‐mode echocardiography images and quantification of cardiac function, including left ventricular end‐diastolic diameter (LVIDd), left ventricular end‐systolic diameter (LVIDs), fractional shortening (FS), and ejection fraction (EF), in HINT3^TG^ and HINT3^NTG^ mice under sham and I/R conditions (n = 10). E) Representative transmission electron microscopy (TEM) images of myocardial ultrastructure in HINT3^TG^ and HINT3^NTG^ mice under sham and I/R conditions at 3000× and 5000× magnifications. Scale bars: 2 µm (3000×) and 0.5 µm (5000×).

To investigate the therapeutic potential of HINT3 in myocardial I/R injury, we generated a cardiomyocyte‐specific HINT3 overexpression mouse model (HINT3^TG^) using the same methodology as described previously (Figure 4A,B; Figure S1F, Supporting Information). Following tamoxifen administration, these mice exhibited cardiomyocyte‐specific overexpression of HINT3. Infarct size was first assessed by TTC staining, which showed that HINT3 overexpression significantly reduced infarct size compared to controls (Figure **4**C). Reducing the infarct size has been shown to be a key determinant of improved cardiac outcomes following I/R injury.^[^ ^25^ ^]^ This finding suggests that increased HINT3 expression mitigates the extent of myocardial damage following I/R injury. Echocardiographic measurements further revealed that overexpression of HINT3 improved cardiac function post‐I/R injury, with a significant reduction in left ventricular internal diameter at systole (LVIDs) and substantial improvements in fractional shortening (FS) and ejection fraction (EF) (Figure 4D), indicating functional protection to the heart during reperfusion. To evaluate the effect of HINT3 overexpression on myocardial injury, we assessed the levels of circulating cardiac injury biomarkers including LDH, CK‐MB, and cTnT in HINT3^TG^ and control mice (HINT3^NTG^) following I/R injury. As shown in (Figure S3A–C, Supporting Information), I/R injury led to a significant increase in serum LDH, CK‐MB, and cTnT levels in both groups. However, HINT3^TG^ mice exhibited markedly reduced levels of all three biomarkers compared to controls, suggesting that HINT3 overexpression alleviates I/R‐induced cardiac injury. To investigate the cellular mechanisms underlying these protective effects, mitochondrial morphology was examined using transmission electron microscopy (TEM). Mitochondrial structural integrity is critical for maintaining ATP production and reducing oxidative stress during reperfusion.^[^ ^26^ ^]^ In HINT3‐overexpressing mice, myocardial mitochondria exhibited better structural integrity, with fewer signs of damage such as disrupted cristae or swollen mitochondria, compared to the control group (Figure 4E). This suggests that HINT3 overexpression may help preserve mitochondrial function during I/R injury.

### 2.5 HINT3 Overexpression Protects Against Cardiomyocyte Apoptosis and Enhances Mitochondrial Function

**Figure 5:**
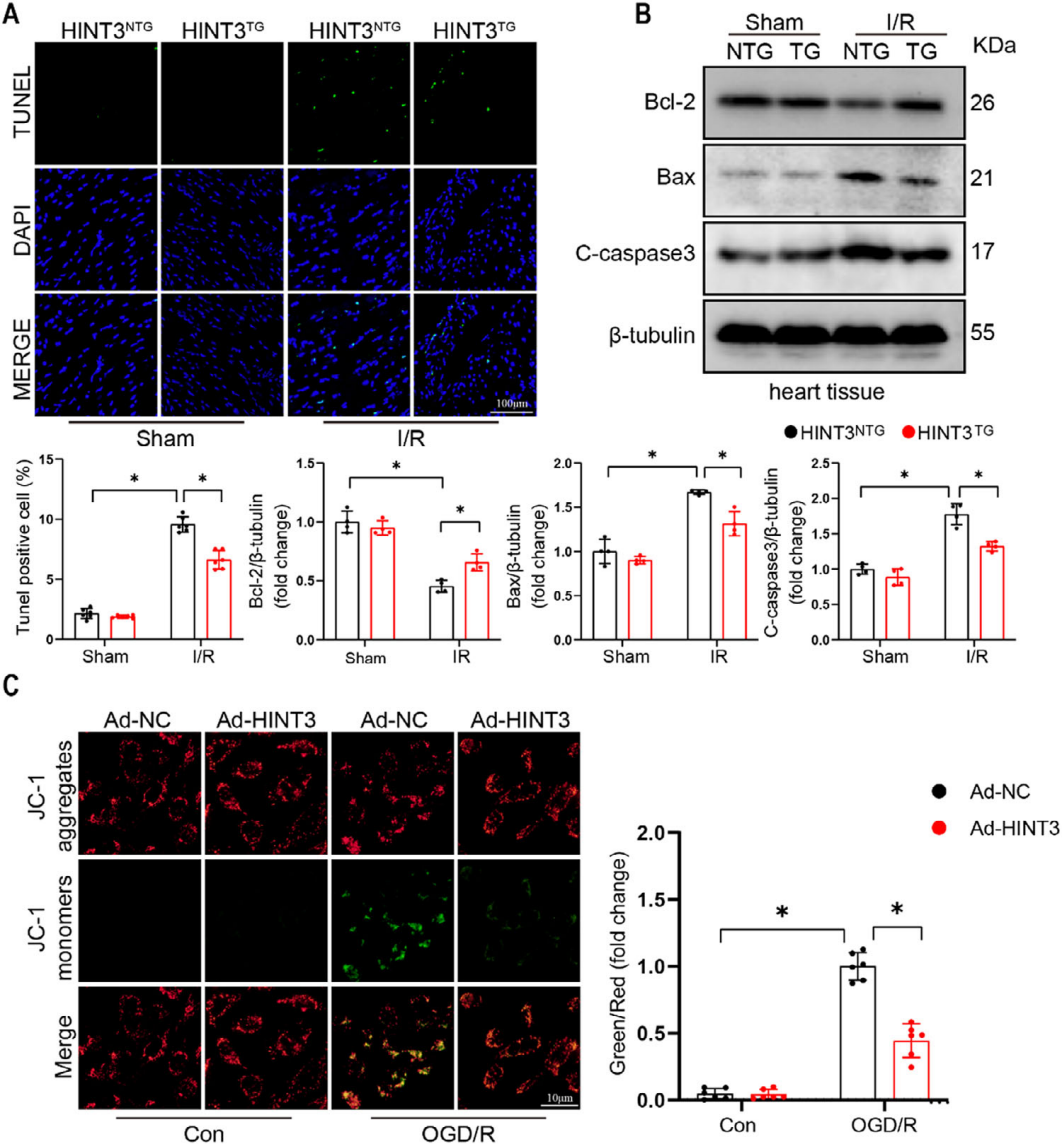
HINT3 overexpression attenuates apoptosis and preserves mitochondrial membrane potential during I/R injury. A) Representative TUNEL staining images showing apoptotic cells (green) and DAPI‐stained nuclei (blue) in cardiac tissue from HINT3^NTG^ and HINT3^TG^ mice under sham and I/R conditions. (n = 6). Scale bar: 100 µm. B) Representative Western blot analysis of apoptotic markers (Bcl‐2, Bax, and cleaved caspase‐3) in cardiac tissue from HINT3^NTG^ and HINT3^TG^ mice under sham and I/R conditions (n = 4). C) Representative JC‐1 staining images showing mitochondrial membrane potential in AC16 cardiomyocytes treated with Ad‐NC or Ad‐HINT3 under control or OGD/R conditions (n = 6), data are presented as fold change relative to OGD/R + Ad‐NC group. Scale bar: 10 µm.

To investigate the role of HINT3 overexpression in I/R injury, we first examined the effect of cardiomyocyte‐specific HINT3 overexpression on apoptosis in heart tissue using TUNEL staining. HINT3 overexpression significantly reduced the number of apoptotic cells induced by I/R injury, suggesting that it protects against cardiomyocyte apoptosis during reperfusion (Figure **5**A). Western blot analysis was then performed to assess the impact of HINT3 overexpression on apoptosis‐related proteins. HINT3 overexpression significantly upregulated the expression of the anti‐apoptotic protein BCL‐2, while markedly decreasing the expression of the pro‐apoptotic proteins BAX and cleaved caspase‐3 (Figure 5B). We observed similar changes in our cellular experiments (Figure S3E‐G, Supporting Information). These results indicate that HINT3 overexpression modulates apoptotic signaling pathways, promoting cell survival during I/R injury. To further evaluate the effects of HINT3 overexpression on mitochondrial function, adenoviral‐mediated HINT3 overexpression was performed in AC16 cells, followed by oxygen‐glucose deprivation and reoxygenation (OGD/R) treatment. Mitochondrial membrane potential (MMP) was assessed using JC‐1 staining. HINT3 overexpression significantly mitigated the reduction in mitochondrial membrane potential induced by OGD/R (Figure 5C). This finding suggests that HINT3 overexpression helps preserve mitochondrial integrity and function during ischemia‐reperfusion injury.

### 2.6 HINT3 Interacts with SDHA to Regulate Mitochondrial Function During I/R Injury

**Figure 6:**
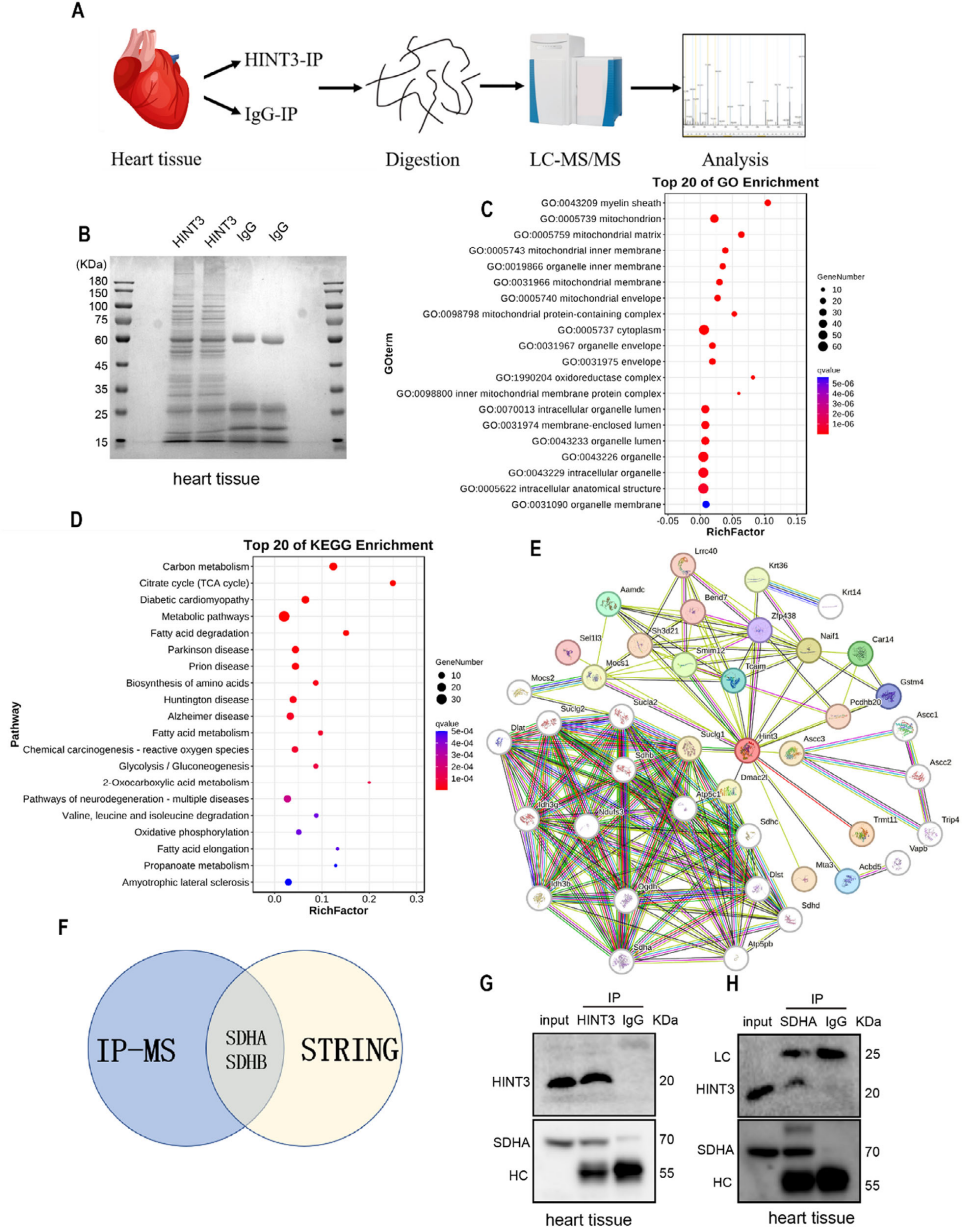
Identification of HINT3‐interacting proteins and validation of HINT3‐SDHA interaction. A) Schematic representation of the experimental workflow for identifying HINT3‐interacting proteins. B) SDS‐PAGE showing protein profiles of immunoprecipitates from heart tissue lysates. C) Bubble chart showing the top 20 significantly enriched Gene Ontology (GO) terms associated with HINT3‐interacting proteins identified by IP‐MS. D) Bubble chart showing the top 20 significantly enriched KEGG pathways associated with HINT3‐interacting proteins. E) Protein‐protein interaction network of HINT3 and its predicted interacting partners, constructed using the STRING database. F) Venn diagram showing overlapping HINT3‐interacting proteins identified by IP‐MS and STRING database predictions. G) Western blot analysis of immunoprecipitates obtained using HINT3‐specific antibodies. Co‐immunoprecipitation confirms that SDHA interacts with HINT3. H) Western blot analysis of immunoprecipitates obtained using SDHA‐specific antibodies. Co‐immunoprecipitation confirms that HINT3 interacts with SDHA.

To investigate the specific molecular mechanisms through which HINT3 improves I/R injury, heart tissue was collected from C57BL/6 mice and immunoprecipitation (IP) was performed using an anti‐HINT3 antibody to pull down proteins interacting with HINT3. The pulled‐down proteins were analyzed by mass spectrometry (MS), revealing potential HINT3‐interacting proteins (Figure **6**A,B). Gene Ontology (GO) and Kyoto Encyclopedia of Genes and Genomes (KEGG) enrichment analyses on the proteins identified by IP‐MS indicated significant associations with the mitochondrial tricarboxylic acid (TCA) cycle and fatty acid degradation pathways (Figure 6C,D). Similarly, the GO analysis identified significant enrichment in mitochondrial components, including the mitochondrial matrix, inner membrane, and mitochondrial membrane (Figure 6C). To further explore potential HINT3 interactors, the STRING database was used for protein‐protein interaction predictions. By intersecting the IP‐MS results with STRING data, two key proteins, SDHA and SDHB, were identified as potential interactors of HINT3 (Figures 6E,F). SDHA and SDHB are catalytic subunits of succinate dehydrogenase (SDH) located in the mitochondrial matrix.^[^ ^27^ ^]^ SDHA catalyzes the oxidation of succinate to fumarate, with electrons transferred through an iron‐sulfur cluster in SDHB to the membrane subunits SDHC and SDHD, where they are ultimately delivered to the electron transport chain (ETC).^[^ ^28^ ^]^ This complex has two distinct enzymatic activities: succinate oxidation (SDH activity) and electron transfer (complex II activity). Recent studies have shown that SDH activity is closely linked to the acetylation modification of SDHA. For example, Myc has been demonstrated to promote acetylation‐dependent inactivation of SDHA by activating SKP2‐mediated degradation of the SIRT3 deacetylase, reducing SDH activity and causing cellular succinate accumulation, which triggers H3K4me3 activation and tumor‐specific gene expression.^[^ ^29^ ^]^ Furthermore, SDHA is the only known acetylated subunit in complex II, and SDH activity correlates with the acetylation level of SDHA.^[^ ^28^ ^]^ During reperfusion, the oxidation of succinate, which accumulates in large amounts during ischemia, is a major contributor to I/R injury. Given these findings, SDHA was selected as a target for further investigation. To validate the interaction between HINT3 and SDHA, co‐immunoprecipitation (Co‐IP) assays were performed. The results confirmed that HINT3 interacts with SDHA (Figure 6G,H). To further confirm whether the interaction between HINT3 and SDHA occurs within mitochondria, we performed Co‐IP using mitochondrial protein fractions isolated from mouse heart tissue. As shown in Figure S4E (Supporting Information), immunoprecipitation with an anti‐HINT3 antibody successfully pulled down SDHA, indicating an interaction between HINT3 and SDHA in the mitochondrial compartment. These findings suggest that HINT3 interacts with SDHA, a key enzyme in the mitochondrial TCA cycle, and may play a crucial role in regulating mitochondrial function during I/R injury.

### 2.7 HINT3 Modulates SDHA Acetylation at K335 and Mitochondrial Function Under OGD/R Conditions

**Figure 7:**
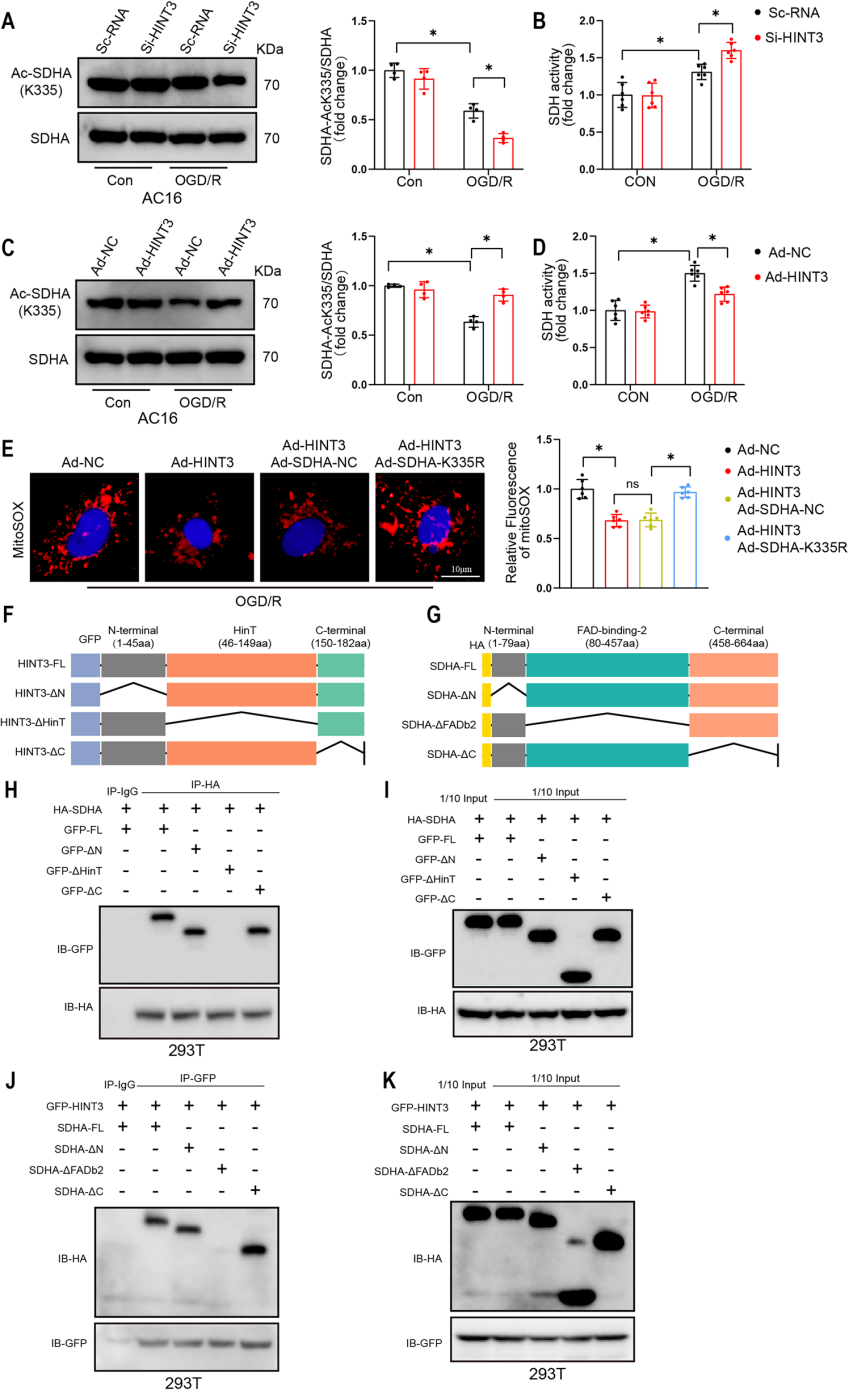
HINT3 regulates SDHA acetylation at K335, affecting SDH activity and mitochondrial ROS production. A) Representative Western blot showing acetylation levels of SDHA at K335 (Ac‐SDHA) in AC16 cardiomyocytes transfected with control (Sc‐RNA) or HINT3 knockdown (Si‐HINT3) under control (Con) and OGD/R conditions (n = 4). B) SDH enzymatic activity in AC16 cardiomyocytes transfected with Sc‐RNA or Si‐HINT3 under control and OGD/R conditions (n = 6). C) Representative Western blot showing Ac‐SDHA levels in AC16 cells infected with control adenovirus (Ad‐NC) or HINT3‐overexpressing adenovirus (Ad‐HINT3) under control and OGD/R conditions (n = 4). D) SDH enzymatic activity in AC16 cells infected with Ad‐NC or Ad‐HINT3 under control and OGD/R conditions (n = 6). E) Mitochondrial ROS levels detected by MitoSOX fluorescence in AC16 cells under OGD/R conditions treated with Ad‐NC, Ad‐HINT3, Ad‐SDHA‐NC, or Ad‐SDHA‐K335R (non‐acetylatable SDHA mutant) (n = 6). Scale bar: 10 µm. F) Schematic diagram of HINT3 full‐length (HINT3‐FL) and deletion mutants, including HINT3‐ΔN (N‐terminal deletion, 1–45aa), HINT3‐ΔHinT (HinT domain deletion, 46–149aa), and HINT3‐ΔC (C‐terminal deletion, 150–182aa), tagged with GFP. G) Schematic diagram of SDHA full‐length (SDHA‐FL) and deletion mutants, including SDHA‐ΔN (N‐terminal deletion, 1–79aa), SDHA‐ΔFADb2 (FAD‐binding domain deletion, 80–457aa), and SDHA‐ΔC (C‐terminal deletion, 458–664aa), tagged with HA. H) Co‐immunoprecipitation (Co‐IP) of HA‐tagged SDHA‐FL with GFP‐tagged HINT3 mutants in HEK293T cells. I) Western blot analysis of inputs and Co‐IP samples from (H), demonstrating successful expression of all GFP‐ and HA‐tagged proteins. J) Co‐IP of GFP‐tagged HINT3‐FL with HA‐tagged SDHA mutants in HEK293T cells. K) Western blot analysis of inputs and Co‐IP samples from (J), confirming successful expression of all GFP‐ and HA‐tagged proteins.

Previous studies have demonstrated that SDH activity is enhanced by the deacetylation of SDHA at K335.^[^ ^29^ ^]^To investigate the relationship between SDHA acetylation at K335 and SDH activity under OGD/R, a specific antibody that detects acetylation at lysine 335 of SDHA was used. Our results revealed that under OGD/R stimulation, knockdown of HINT3 significantly reduced acetylation at K335 of SDHA, leading to a marked increase in SDH enzyme activity. Conversely, overexpression of HINT3 resulted in higher acetylation levels at K335, which was associated with a decrease in SDH activity (Figure **7**A‐D). To explore the effect of SDHA deacetylation on mitochondrial ROS production, an adenoviral vector mimicking the deacetylated form of SDHA at K335 was constructed. MitoSOX staining was used to measure mitochondrial ROS levels under OGD/R conditions. The data demonstrated that mimicking SDHA deacetylation at K335 reversed the decrease in mitochondrial ROS levels observed upon HINT3 overexpression (Figure 7E). In cellular experiments, we also found that mimicking the deacetylation mutation of the SDHA K335 site reversed the increase in mitochondrial membrane potential induced by HINT3 overexpression (Figure S4A,B, Supporting Information). To further investigate the molecular mechanism underlying these observations, structural domain deletion mutants of both HINT3 and SDHA were generated (Figure 7F,G). Co‐IP assays demonstrated that the HinT domain (46‐149 aa) of HINT3 strongly interacts with the FAD‐binding‐2 domain (80‐457 aa) of SDHA, which includes the K335 site. This interaction may suggests that HINT3 directly modulates SDHA acetylation at K335, thereby influencing SDH activity (Figure 7H‐K). To determine whether the regulatory effect of HINT3 on SDHA acetylation is dependent on its enzymatic activity, we introduced a point mutation (H145A) that disrupts the putative catalytic site of HINT3. Co‐immunoprecipitation assays in AC16 cells showed that the H145A mutant retained its ability to bind SDHA, comparable to wild‐type HINT3 (Figure S4C, Supporting Information), indicating that the interaction between HINT3 and SDHA is independent of HINT3 enzymatic activity. Furthermore, under OGD/R conditions, overexpression of the H145A mutant HINT3 suppressed SDHA K335 acetylation to a similar extent as wild‐type HINT3 (Figure S4D, Supporting Information), suggesting that HINT3 regulates SDHA acetylation through a non‐enzymatic mechanism, possibly via steric hindrance or competitive binding. These findings provide crucial insights into the molecular mechanisms by which HINT3 regulates SDH activity and mitochondrial function. The interaction between the HinT domain of HINT3 and SDHA's FAD‐binding domain may represent a key regulatory mechanism for SDHA acetylation and, consequently, mitochondrial ROS production during I/R injury.

### 2.8 HINT3 and HDAC1 Form a Regulatory Axis Modulating SDHA Acetylation and Cardiomyocyte Apoptosis

**Figure 8:**
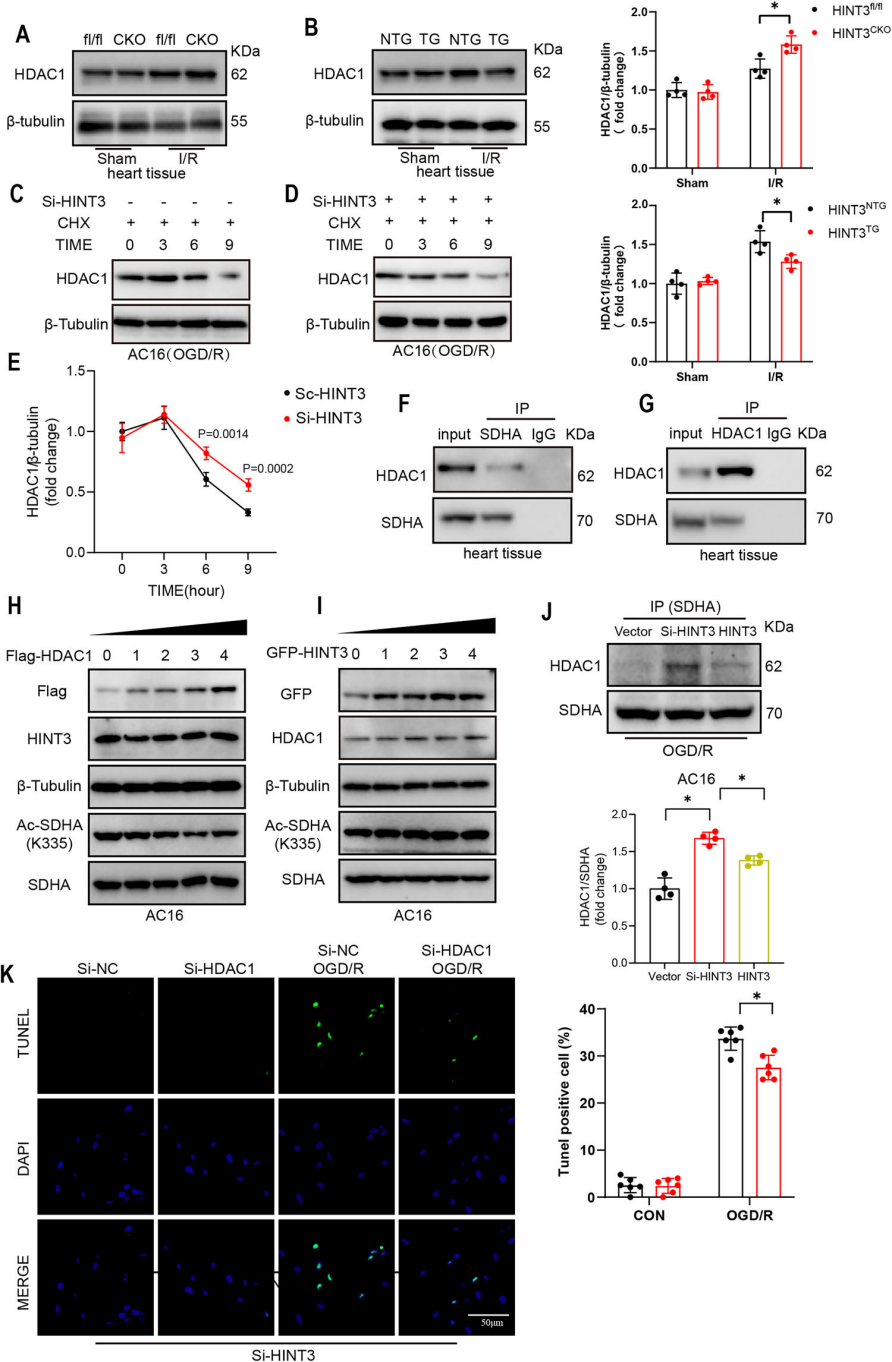
HINT3 regulates HDAC1 expression and competes with HDAC1 for SDHA binding in a disease‐dependent manner. A) Representative Western blot showing HDAC1 expression in heart tissues from HINT3^fl/fl^ and HINT3^CKO^ mice under sham and I/R conditions (n = 4). B) Representative Western blot showing HDAC1 expression in heart tissues from HINT3^NTG^ and HINT3^TG^ mice under sham and I/R conditions (n = 4). C–E) CHX chase assay in AC16 cells under OGD/R conditions, showing HDAC1 protein stability after HINT3 knockdown (n = 4). F,G) Co‐IP showing interaction between SDHA and HDAC1 in mouse heart tissue. H,I) Western blots of SDHA K335 acetylation in AC16 cells with gradient overexpression of Flag‐HDAC1 (H) or GFP‐HINT3 (I) under normoxia. J) Co‐IP of SDHA‐bound HDAC1 in AC16 cells under OGD/R with control, HINT3 knockdown, or overexpression (n = 4). K) TUNEL staining of AC16 cells under control or OGD/R conditions with HINT3 knockdown and HDAC1 knockdown (n = 6). Scale bar: 50 µm.

Post‐translational modifications (PTMs), such as acetylation, SUMOylation, and ubiquitination, play critical roles in regulating SDH activity.^[^ ^30^, ^31^, ^32^ ^]^ Previous studies have shown that the interaction between SDHA and HDAC1 increases mitochondrial ROS levels during reperfusion by deacetylating SDHA at K335.^[^ ^33^ ^]^ Also, Myc has been demonstrated to promote acetylation‐dependent inactivation of SDHA by activating SKP2‐mediated degradation of the SIRT3 deacetylase, reducing SDH activity.^[^ ^29^ ^]^ Based on this, we investigated how HINT3 modulates HDAC1 and SIRT3 expression and function under I/R injury conditions. Our results showed that SIRT3 expression was not significantly different between HINT3^fl/fl^ and HINT3^CKO^ mice under either sham conditions (Figure S5A, Supporting Information) or following I/R injury (Figure S5B, Supporting Information). However, HDAC1 expression was significantly upregulated in cardiac tissue under I/R injury. HINT3 knockout further enhanced HDAC1 expression, while HINT3 overexpression reversed this increase (Figure **8**A,B). These findings indicate that HINT3 regulates HDAC1 levels under I/R conditions. To determine the subcellular localization of HDAC1 in heart tissue, we fractionated nuclear, cytoplasmic, and mitochondrial proteins and performed Western blot analysis. As shown in Figure S5C (Supporting Information), HDAC1 was detected in all three compartments, with the highest abundance observed in the nuclear fraction. This finding suggests that HDAC1 is not exclusively nuclear, but also partially distributed in the cytoplasm and mitochondria, supporting its potential role in modulating mitochondrial protein acetylation under stress conditions. To further elucidate the mechanism by which HINT3 regulates HDAC1 expression, we analyzed transcriptome data from cardiac tissues of HINT3^CKO^ and HINT3^fl/fl^ mice following I/R injury. The results showed no significant difference in HDAC1 mRNA levels between the two groups (Figure S5E, Supporting Information), suggesting that HINT3 does not regulate HDAC1 expression at the transcriptional level. To investigate whether HINT3 affects HDAC1 protein stability under ischemic stress, we performed Cycloheximide chase experiment in AC16 cells subjected to OGD/R. In control cells, HDAC1 protein levels gradually decreased over time after CHX treatment, indicating normal degradation dynamics under OGD/R conditions (Figure 8C,E). However, in HINT3‐silenced cells, HDAC1 degradation was significantly attenuated (Figure 8D,E), suggesting that loss of HINT3 stabilizes HDAC1 protein under OGD/R stress. These findings demonstrate that HINT3 promotes HDAC1 degradation in cardiomyocytes following ischemic injury, and this regulatory effect occurs at the post‐translational level.

To further confirm the interaction between SDHA and HDAC1, Co‐IP assays were performed, which confirmed that HDAC1 interacts with SDHA (Figure 8F,G). Given that the region of SDHA interacting with HINT3 includes the acetylation site at K335, and that HINT3 modulates acetylation at this site, we hypothesized that HINT3 competes with HDAC1 for binding to SDHA. To test this hypothesis, we incrementally overexpressed either HINT3 or HDAC1 in AC16 cells and examined their effects on SDHA acetylation at K335. Under normoxic culture conditions, we performed a gradient overexpression of HDAC1 in AC16 cells and observed a corresponding decrease in SDHA‐K335 acetylation levels (Figure 8H), indicating that HDAC1 negatively regulates SDHA acetylation in a dose‐dependent manner. Next, to assess whether HINT3 could directly affect SDHA acetylation, we overexpressed HINT3 in a similar gradient manner. Interestingly, despite increasing HINT3 levels, no significant change in SDHA‐K335 acetylation was detected (Figure 8I), consistent with earlier results (Figure 7A,C) showing that alterations in HINT3 expression under basal conditions do not affect SDHA acetylation. This is likely due to the fact that HDAC1 protein levels remain unaffected by HINT3 under normal conditions, thereby maintaining stable deacetylation activity toward SDHA. These findings suggest that the regulatory role of HINT3 in modulating HDAC1 and SDHA acetylation is disease‐context dependent, becoming functionally relevant only under pathological conditions such as I/R or OGD/R injury. Finally, we evaluated the interaction dynamics between SDHA and HDAC1 under OGD/R conditions. Co‐immunoprecipitation using anti‐SDHA antibodies revealed that knockdown of HINT3 significantly enhanced the amount of HDAC1 co‐precipitated with SDHA (Figure 8J), indicating that HINT3 depletion facilitates HDAC1 binding to SDHA. Collectively, these results suggest that HINT3 regulates SDHA acetylation indirectly by modulating HDAC1 availability in a disease‐context‐dependent manner, likely through competitive binding to SDHA rather than by direct enzymatic action.

To investigate the functional consequences of HDAC1 inhibition on mitochondrial ROS production, HDAC1 expression was silenced in AC16 cardiomyocytes under OGD/R conditions. MitoSOX staining revealed that HDAC1 knockdown reversed the mitochondrial ROS increase observed with HINT3 knockdown (Figure S5D, Supporting Information). Furthermore, TUNEL staining showed that HDAC1 silencing significantly attenuated cardiomyocyte apoptosis induced by HINT3 knockdown under OGD/R conditions (Figure 8K). These findings suggest that HINT3 and HDAC1 form a regulatory axis that modulates SDHA acetylation, mitochondrial ROS production, and cardiomyocyte apoptosis under I/R stress.

To further assess the long‐term impact of HINT3 deficiency after I/R injury, we established a chronic I/R model with 14 days of reperfusion. HINT3^CKO^ mice exhibited significantly reductions in cardiac function and increased myocardial fibrosis compared to HINT3^fl/fl^ controls. These findings suggest that loss of HINT3 exacerbates adverse cardiac remodeling and dysfunction in the chronic phase post‐I/R injury (Figure S6, Supporting Information).

## 3 Discussion

In this study, we experimentally confirmed the protective role of HINT3 in cardiac I/R injury. Our results demonstrated that cardiomyocyte‐specific knockout of HINT3 significantly exacerbated the decline in cardiac function and infarct size caused by I/R injury. Furthermore, apoptosis levels in cardiac cells were significantly increased, mitochondrial swelling was observed, and mitochondrial cristae were more severely disrupted. Additionally, HINT3 knockout in the I/R injury model modulated key apoptosis‐related proteins and decreased mitochondrial membrane potential, thus significantly worsening the I/R injury. In contrast, cardiomyocyte‐specific overexpression of HINT3 significantly alleviated these cellular damage. Mechanistically, we discovered that HINT3 interacts with SDHA and regulates the enzyme activity of the SDH complex, which further impacts mitochondrial ROS production. Specifically, HINT3 increased the acetylation level of SDHA at the K335 site, thereby inhibiting SDH enzyme activity. This process plays a crucial protective role in cardiac I/R injury. Overall, our study demonstrates that HINT3 significantly mitigates cardiac I/R injury by regulating mitochondrial morphology and function, apoptosis, and ROS production. These findings provide important evidence for HINT3 as a potential therapeutic target for cardiac protection and offer novel insights for the treatment of cardiac I/R injury.

Mitochondrial damage plays a crucial role in the development and progression of cardiovascular diseases.^[^ ^34^, ^35^, ^36^ ^]^ In recent years, studies on the HINT protein family in cardiovascular diseases have consistently pointed to mitochondria. Gao et al. reported that HINT1 was significantly reduced in muscle tissue of STZ‐induced diabetic mice and high‐glucose (HG)‐treated endothelial cells (EC). The absence of HINT1 impaired blood flow recovery and angiogenesis, while HINT1 deficiency exacerbated mitochondrial dysfunction in ECs, including impaired mitochondrial respiration, reduced mitochondrial membrane potential, and increased ROS levels.^[^ ^37^ ^]^ Similarly, Li et al. found that in myocardial I/R injury models and OGD/R models, HINT2 expression was reduced in damaged cardiac tissue and cardiac microvascular endothelial cells. Overexpression of HINT2 inhibited the MCU complex‐mitochondrial calcium overload‐mitochondrial fission and apoptosis pathways, thereby alleviating I/R injury in the cardiac microvascular endothelium.^[^ ^38^ ^]^ We also found that HINT2 expression was lower in failing hearts and hypertrophic cardiomyocytes. Studies suggest that HINT2 mitigates pressure‐overload‐induced cardiac remodeling by affecting the activity and assembly of mitochondrial complex I.^[^ ^14^ ^]^ However, research on HINT3, a family member of HINT1 and HINT2, is scarce, especially in the context of cardiovascular diseases. It remains unclear whether HINT3, like its family members, contributes to the pathogenesis of cardiovascular diseases or participates in mitochondrial regulation. By constructing a mouse myocardial I/R injury model and OGD/R model, we have expanded the understanding of HINT3 in cardiovascular diseases and mitochondrial function. Consistent with the aforementioned studies, we observed a reduction in HINT3 protein expression in both the mouse myocardial I/R injury model and the OGD/R model. Cardiomyocyte‐specific knockout of HINT3 worsened I/R‐induced cardiac dysfunction and increased infarct size. It also exacerbated mitochondrial swelling and the loss of mitochondrial cristae in myocardial tissue. Our in vitro experiments (AC16 cardiomyocytes) further confirmed HINT3's protective effect, as HINT3 overexpression prevented the loss of mitochondrial membrane potential induced by OGD/R. In addition to its protective effects on mitochondrial morphology and function, RNA‐Seq analysis revealed that the expression of apoptosis‐related genes in myocardial tissue from HINT3 knockout mice was significantly altered in the myocardial I/R injury model. The absence of HINT3 exacerbated I/R‐induced cell apoptosis. Over the past two decades, the involvement of apoptosis in I/R injury has been well recognized.^[^ ^39^, ^40^ ^]^ Myocardial cell apoptosis occurs via intrinsic pathways in response to DNA damage and increased ROS and cytosolic Ca^2+^ levels, or through extrinsic pathways in response to activation of death receptors on the cell membrane. Apoptosis requires energy and involves mitochondrial release of cytochrome c and activation of caspases, leading to typical DNA fragmentation.^[^ ^41^ ^]^ Li et al. recently reported that HINT3 inhibits the activation of the PTEN/AKT/mTOR signaling pathway, suppressing the proliferation, growth, migration, and tumor development of MCF‐7 and MDA‐MB‐231 breast cancer cells. Knockdown of HINT3 significantly reduced apoptosis in MCF‐7 and MDA‐MB‐231 cells, whereas overexpression of HINT3 significantly promoted apoptosis in both cell lines.^[^ ^11^ ^]^ This discrepancy may be attributed to tissue‐specific and physiological differences. Breast cancer cells (such as MCF‐7 and MDA‐MB‐231) have distinct biological characteristics and metabolic demands compared to cardiomyocytes. HINT3 may exert different functions through various mechanisms depending on the cell type.

Our IP‐MS results showed that the proteins interacting with HINT3 were enriched in the mitochondrial matrix and inner mitochondrial membrane, which is similar to other members of the HINT protein family.^[^ ^14^, ^37^, ^38^ ^]^ This suggests that HINT3 may also participate in I/R injury by regulating mitochondrial function. Specifically, we identified SDHA, a subunit of the SDH enzyme complex, as a key candidate to explore the specific mechanism of HINT3. SDH and succinate are strongly interconnected in the TCA cycle and the electron transport chain (ETC). Known as Complex II, SDH plays a critical role in the integration of two major mitochondrial pathways: the oxidation of succinate to fumarate as an essential component of the TCA cycle and the conversion of ubiquinone to ubiquinol in the mitochondrial ETC. Both of these processes are indispensable for oxidative phosphorylation, which is responsible for ATP production and sustaining biosynthesis.^[^ ^42^, ^43^ ^]^ Under ischemic conditions, hypoxia reduces mitochondrial respiration and leads to an accumulation of succinate. Subsequent reperfusion promotes SDH to rapidly oxidize the accumulated succinate. Reverse electron transfer (RET) via mitochondrial Complex I allows some electrons to escape from the ETC, causing insufficient oxygen reduction reactions, thereby driving the production of ROS, which leads to oxidative damage to cellular components such as lipids, proteins, and DNA.^[^ ^7^ ^]^ SDH is a key enzyme in the formation of succinate during ischemia and its oxidation during reperfusion. In recent years, increasing studies have found that malonate, a competitive SDH inhibitor, has become a potential therapy to selectively inhibit SDH and reduce reperfusion injury. For example, Mottahedin et al. found in a stroke model that the SDH inhibitor malonate reduces acute brain injury in a dose‐dependent manner during reperfusion by targeting succinate oxidation.^[^ ^19^ ^]^ Because the action of malonate depends on the local pH decrease during ischemia, which facilitates selective uptake by high‐risk tissues via MCT1 during reperfusion,^[^ ^2^ ^]^ Abe et al. found that the administration of acidified malonate during reperfusion enabled low‐dose malonate to provide long‐term cardiac protection after myocardial infarction, thus preventing IRI and heart failure following MI.^[^ ^20^ ^]^ In our in vitro experiments, knockdown of HINT3 led to an increase in SDH enzymatic activity, whereas overexpression of HINT3 resulted in a decrease in SDH activity. Notably, these effects were observed specifically under OGD/R‐induced stress conditions, but not under normal culture conditions, indicating that the regulatory role of HINT3 on SDH activity is disease‐context dependent. This modulation was associated with changes in the acetylation level of SDHA at the K335 site, a modification known to influence SDH activity, consistent with previous findings by Li et al.^[^ ^29^ ^]^ By constructing deletion mutants of HINT3 and SDHA proteins to explore the structural domains involved in their interaction, we found that HINT3 interacts with the FAD‐binding‐2 domain of SDHA, which precisely contains the K335 site. HINT3, as a nucleotide‐binding and hydrolyzing DNA repair enzyme, likely regulates the acetylation of SDHA through the action of other acetylation‐modifying enzymes. In Parkinson's disease, downregulation of SIRT3 leads to hyperacetylation of SDHA, resulting in reduced SDH activity and decreased ATP production.^[^ ^44^ ^]^ Herr et al. discovered that HDAC1 binds to SDHA, and inhibition of HDAC1 during reperfusion reduces SDHA activity, thus alleviating myocardial reperfusion injury in the early stages of reperfusion.^[^ ^33^ ^]^ We also verified this phenomenon through Co‐IP, and we found that HDAC1 expression was upregulated in HINT3 cardiomyocyte‐specific knockout mice, while overexpression of HINT3 led to reduced HDAC1 levels. Notably, this regulatory effect was observed specifically under I/R conditions, but not under sham conditions, where HDAC1 expression remained unchanged regardless of HINT3 levels. Through transcriptomic analysis and Cycloheximide chase experiment (CHX), we demonstrated that HINT3 modulates HDAC1 expression by influencing its protein stability, rather than transcription. However, the exact molecular mechanism by which HINT3 promotes HDAC1 degradation remains to be determined, and further studies will be required to clarify this pathway.

Protein‐protein interactions often influence their post‐translational modifications. Huang et al. discovered that C4orf19 binds to the DGR domain of Keap1, blocking the ubiquitination sites of Keap1 from interacting with TRIM25, thereby inhibiting Keap1 degradation.^[^ ^45^ ^]^ To probe how HINT3 and HDAC1 affect SDHA acetylation at K335, we performed gradient overexpression experiments under normal conditions. We found that raising HDAC1 levels led to a marked decrease in SDHA‐K335 acetylation, whereas raising HINT3 levels had no appreciable effect (Figure 8H,I), consistent with our earlier observation that HINT3 alone does not alter SDHA acetylation in the absence of stress. Importantly, while HDAC1 is a downstream effector of HINT3, its activity toward SDHA appears not to be disease‐context dependent, as its overexpression alone is sufficient to reduce SDHA acetylation. In contrast, HINT3‐mediated regulation of HDAC1 and SDHA acetylation occurs only under pathological conditions such as OGD/R or I/R. To further clarify whether HINT3 and HDAC1 compete for SDHA binding under disease conditions, we conducted a co‐immunoprecipitation assay using SDHA antibody in OGD/R‐treated cells. The results revealed that knockdown of HINT3 significantly increased the binding of HDAC1 to SDHA (Figure 8J), indicating that HINT3 competes with HDAC1 for SDHA binding in a disease‐dependent manner. Collectively, these findings suggest a context‐specific regulatory mechanism in which HINT3 interferes with HDAC1‐mediated deacetylation of SDHA during ischemic stress by blocking HDAC1 access to the K335 site, rather than altering acetylation directly under normal conditions. This may reflect a latent regulatory mechanism that is only activated under pathological stress. Under conditions of HINT3 inhibition, simultaneous inhibition of HDAC1 expression reversed the mitochondrial ROS surge induced by HINT3 knockdown under OGD/R stimulation and decreased SDH enzyme activity.

Our study is limited using exclusively male mice. There is clear evidence that gender‐related factors and their interactions lead to differences in cardiovascular disease outcomes between males and females, and may even produce opposite effects on clinical manifestations and outcomes. Biological differences typically favor females in the presentation of cardiovascular diseases. Conversely, in socio‐cultural contexts, the association between psychological stress and disease manifestation is stronger in females, and there is relatively less drug development targeting the female population, resulting in greater adverse impacts on females compared to males.^[^ ^46^ ^]^ In future studies, we will explore the potential differential roles of cardiac HINT3 in female myocardial ischemia‐reperfusion injury.

## 4 Conclusion

In conclusion, our study elucidates the pivotal protective role of HINT3 in cardiac I/R injury. We demonstrated that cardiomyocyte‐specific knockout of HINT3 significantly exacerbates cardiac dysfunction, increases infarct size, and intensifies mitochondrial damage and apoptosis. Conversely, overexpression of HINT3 markedly protects the heart from these adverse effects. Mechanistically, HINT3 interacts with the mitochondrial enzyme SDHA, enhancing its acetylation at the K335 site, thereby inhibiting SDH activity and subsequent reactive oxygen species (ROS) production. This interaction effectively mitigates mitochondrial oxidative stress, preserves mitochondrial integrity, and contributes to reduced apoptosis and improved cardiac function during I/R injury. Our findings position HINT3 as a promising therapeutic target. Strategies to pharmacologically enhance HINT3 activity or to modulate its downstream effectors SDHA and HDAC1 could represent novel approaches to treat cardiac dysfunction caused by I/R injury.

## 5 Experimental Section

### Animal Models

All animal experiments were conducted in accordance with the guidelines of the Animal Welfare Ethics Committee at Renmin Hospital of Wuhan University (No. WDRM20190906). This study was conducted in accordance with the National Institutes of Health's Guidelines for the Care and Use of Laboratory Animals (National Institutes of Health Publication, revised 2011). Male C57BL/6 mice (8‐12 weeks old) were obtained from the Institute of Laboratory Animal Science, Chinese Academy of Medical Sciences (Beijing, China).

HINT3^flox/flox^ mice were purchased from GemPharmatech Co., Ltd. To generate cardiomyocyte‐specific HINT3 knockout (HINT3^CKO^) mouse models, HINT3^fl/fl^ mice were mate with Cre recombinase‐expressing mice (αMHC‐Cre). Tamoxifen was administered via daily intraperitoneal injections at a dose of 20 mg/kg/d for five consecutive days to induce Cre‐mediated recombination, as previously described.^[^ ^47^ ^]^ The wildtype (WT) littermates (HINT3^flox/flox^) were kept as control.

HINT3 transgenic mice (HINT3^Rosa26^) were purchased from Shanghai Model Organisms Center, Inc. The CAG‐LSL‐HINT3‐WPRE‐PA expression cassette was precisely inserted into the Rosa26 gene locus through homologous recombination. To generate cardiomyocyte‐specific HINT3 overexpress (HINT3^TG^) mouse models, HINT3^Rosa26^ mice were mate with Cre recombinase‐expressing mice (αMHC‐Cre). Tamoxifen was administered via daily intraperitoneal injections at a dose of 20 mg/kg/d for five consecutive days to induce Cre‐mediated recombination, as previously described. The wildtype (WT) littermates (HINT3^Rosa26^/HINT3^NTG^) were kept as control.

All animal experiments were conducted using male mice to eliminate the influence of sex differences. The animals were housed under specific pathogen‐free (SPF) conditions at temperatures of 20–25 °C and humidity levels of 45–55%, maintained on a standard 12‐h light/dark cycle.

### Induction of Ischemia/Reperfusion (I/R) Injury

Myocardial I/R injury was induced in mice using the left anterior descending (LAD) coronary artery ligation method. Mice were anesthetized with isoflurane (3%) and intubated for mechanical ventilation. A thoracotomy was performed, and the LAD coronary artery was ligated with a 7‐0 silk suture for 45 min suture over a piece of PE‐10 tubing to induce ischemia, followed by reperfusion by releasing the ligature. Sham‐operated mice underwent the same surgical procedure without LAD ligation. Post‐surgery, mice were monitored until recovery and then maintained under standard conditions.^[^ ^48^ ^]^

### TTC‐EB Staining for Infarct Size Assessment

After 24 h of reperfusion, the mice were anesthetized, and a 2% Evans Blue dye solution was perfused through the ascending aorta at a constant flow rate. Subsequently, the hearts were harvested and placed in ice‐cold phosphate‐buffered saline (PBS) to preserve tissue integrity. The hearts were secured in a heart slicer or appropriate holder, and using a sharp blade, transverse sections were cut into 2 mm thick slices. Uniform slicing ensured consistent staining and accurate assessment of infarct size. The heart slices were then immersed in a 2% triphenyltetrazolium chloride (TTC) solution and incubated at 37 °C for 15 min. During this incubation period, viable myocardial cells with active dehydrogenases reduced TTC to form a deep red formazan precipitate, staining the viable tissue red, while infarcted (necrotic) tissue remained pale or unstained. Finally, the TTC‐stained heart slices were transferred to 10% neutral buffered formalin for 15 min to fix the tissues and terminate the staining reaction.^[^ ^49^ ^]^ Infarct size was quantified using ImageJ software by calculating the infarct area (IF) relative to the area at risk (AAR), expressed as the IF/AAR ratio.

### Echocardiography

Cardiac function was evaluated using transthoracic echocardiography with a Vevo® 3100 high‐resolution Preclinical Imaging System (FUJIFILM VisualSonics, Toronto, Canada) equipped with a 30‐MHz linear ultrasound transducer (MX 400) as it recently described under isoflurane anesthesia (1.5%).^[^ ^50^ ^]^ Two‐dimensional guided M model images crossing the anterior/posterior wall of the left ventricle were recorded to measure the LVIDd, LVIDs, and HR from at least five consecutive cardiac cycles.

### Transmission Electron Microscopy (TEM)

Heart was harvested and cut into 1 mm^3^ section from the apical of the left ventricle, then assessed using transmission electron microscopy. They were then fixed in Karnovsky's fixative: 2% Glutaraldehyde and 4% Formaldehyde in 0.1M Sodium Cacodylate (pH 7.4) for 1 h, then incubated at 4 °C overnight. The heart samples were then post‐fixed and filmed, and the images were collected using a Tecnai‐20 electron microscopy (Philips‐FEI, Hillsboro, Oregon). Magnification of ×3000, ×5000, ×8000 and ×12000 were captured, and the mitochondrial morphology were assessed.

### Cell Isolation and Oxygen‐Glucose Deprivation/Reoxygenation (OGD/R) Treatment—Heart Extraction

Neonatal rat cardiomyocytes (NRCMs) and cardiac fibroblasts (NRCFs) were isolated from 1‐ to 3‐day‐old Sprague–Dawley rat pups under sterile conditions. Briefly, the pups were wiped with 75% ethanol and placed in a sterile container. Hearts were rapidly excised through a thoracotomy using ophthalmic scissors, with care taken to minimize contamination. The excised hearts were transferred into pre‐cooled DMEM/F12 medium (10 mL) in a 100‐mm dish and rinsed gently to remove excess blood. Atria and aorta were discarded, and the ventricles were minced into 1‐ to 2‐mm^3^ fragments using ophthalmic scissors. All steps were conducted on ice to maintain cell viability.

### Cell Isolation and Oxygen‐Glucose Deprivation/Reoxygenation (OGD/R) Treatment—Tissue Digestion

The minced tissue fragments were transferred into a glass serum bottle containing 8 mL of digestion solution (0.125% trypsin in D‐Hanks solution) and digested at 37 °C on a magnetic stirrer (150 rpm) for 15 min. The supernatant from the first digestion, containing red blood cells and debris, was discarded. The tissue was further digested with fresh enzyme solution (8 mL) for four 15‐min cycles. After each cycle, the supernatant was collected and added to a 50‐mL centrifuge tube containing DMEM/F12 medium supplemented with 20% fetal bovine serum (FBS) to neutralize the enzyme activity. The collected supernatant was centrifuged at 1500 rpm for 8 min, and the cell pellets were resuspended in an appropriate volume of culture medium.

### Cell Isolation and Oxygen‐Glucose Deprivation/Reoxygenation (OGD/R) Treatment—Differential Adhesion to Separate Cardiomyocytes and Fibroblasts

The cell suspension was passed through a 40‐µm cell strainer to remove cell clumps and transferred to gelatin‐coated 100‐mm culture dishes for differential adhesion. The dishes were incubated for 90 min at 37 °C in a humidified atmosphere with 5% CO_2_ to allow fibroblasts to adhere. Non‐adherent cells (cardiomyocytes) were collected, filtered, and counted using Trypan blue staining. The cardiomyocyte suspension was adjusted to a concentration of 2.5–3 × 10⁵ cells/mL and plated onto six‐well plates precoated with 0.1% gelatin. Bromodeoxyuridine (BrdU, final concentration 0.1 mM) was added to the medium to inhibit the growth of non‐cardiomyocytes. Plates were gently shaken in a north‐south and east‐west direction to distribute the cells evenly.

### Cell Isolation and Oxygen‐Glucose Deprivation/Reoxygenation (OGD/R) Treatment—Culture of Cardiac Fibroblasts

The adherent fibroblasts remaining in the original dishes were cultured in DMEM/F12 medium containing 10% FBS. The medium was changed every 48 h, and cells were passaged as needed.

For OGD/R treatment, cells were subjected to oxygen‐glucose deprivation by incubating them in glucose‐free Dulbecco's modified Eagle's medium/F12 under hypoxic conditions (1% O_2_, 5% CO_2_) for 4 h, followed by reoxygenation by returning to normoxic conditions (21% O_2_, 5% CO_2_) with complete media for 24 h.

### Cell Culture and OGD/R Model in AC16 Cells

The AC16 human cardiomyocyte cell line was cultured in Dulbecco's modified Eagle's medium/F12 supplemented with 10% FBS and 1% penicillin‐streptomycin. For OGD/R experiments, AC16 cells were subjected to oxygen‐glucose deprivation by incubating them in glucose‐free DMEM/F12 under hypoxic conditions (1% O_2_, 5% CO_2_) for 4 h, followed by reoxygenation with complete media under normoxic conditions (21% O, 5% CO_2_) for 24 h.

### Immunofluorescence Staining

Immunofluorescence staining was performed on both cultured cells and heart tissue sections. Cells were fixed with 4% paraformaldehyde, permeabilized with 1% Triton X‐100, and blocked with 10% goat serum for 1 h at room temperature. Primary antibodies against HINT3 and other relevant proteins were incubated overnight at 4 °C. After washing, cells were incubated with fluorescently labeled secondary antibodies (goat anti‐mouse IgG Alexa Fluor 488 or goat anti‐rabbit IgG Alexa Fluor 568 1:200 dilution) for 1 h at room temperature. Nuclei were counterstained with DAPI (1 µg mL^−1^). Fluorescence intensity was quantified using ImageJ software.

### RNA Sequencing (RNA‐seq)

Total RNA was extracted from cardiac tissues of HINT3^fl/fl^ and HINT3^CKO^ mice following ischemia/reperfusion (I/R) injury using Trizol (Life Technologies). RNA quality was assessed using an Agilent Bioanalyzer 2100. mRNA was enriched using mRNA Capture Beads magnetic beads. After magnetic bead purification, the mRNA was fragmented at high temperatures. Using the fragmented mRNA as a template, the first strand of cDNA was synthesized within a reverse transcriptase mixture. Concurrently with the synthesis of the second cDNA strand, end repair was performed and an A‐tail was added. Subsequently, adapters were ligated, and the target fragments were purified using Hieff NGS® DNA Selection Beads magnetic beads. PCR library amplification was then carried out, and the libraries were sequenced using the Illumina Novaseq X Plus platform to generate paired‐end reads. Libraries were prepared using the Hieff NGS® Ultima Dual‐mode mRNA Library Prep Kit (12309ES, Yeasen) and sequenced on the Illumina Novaseq X Plus platform. Differential gene expression analysis was performed using DESeq2, with genes showing fold change > 1.5 and adjusted p‐value < 0.05 considered significant.

### Western Blot Analysis

Protein samples were extracted from cardiac tissues and cultured cells using RIPA buffer supplemented with protease and phosphatase inhibitors. Protein concentrations were determined using the BCA assay. Equal amounts of protein were separated by 10% SDS‐PAGE and transferred to PVDF membranes. Membranes were blocked with 5% non‐fat milk for 1 h at room temperature and incubated with primary antibodies against HINT3, BCL‐2, BAX, cleaved caspase‐3, HDAC1, SDHA, Acetyl‐SDHA‐K335 and *β*‐tubulin HA‐Tag GFP‐tag IgG overnight at 4 °C. After washing, membranes were incubated with HRP‐conjugated secondary antibodies for 1 h at room temperature. Protein bands were visualized using an ECL detection system(ChemiDoc XRS + system) and quantified using ImageJ software.

### Co‐Immunoprecipitation (Co‐IP)

For tissue‐based experiments, heart tissues were harvested from mice and homogenized in RIPA lysis buffer to extract total protein. Equal amounts of protein lysates were used for immunoprecipitation of HINT3 with SDHA and SDHA with HDAC1 using their respective primary antibodies and Protein A/G agarose beads (Beyotime, China). The immunoprecipitated complexes were washed thoroughly, eluted, and subjected to SDS‐PAGE followed by Western blot analysis to confirm the interactions. For cell‐based experiments, HEK293T cells were co‐transfected with GFP‐ or HA‐tagged full‐length and truncated mutant constructs of HINT3 and SDHA to identify the specific domains required for interaction. After 48 h, cells were lysed in RIPA buffer, and lysates were subjected to immunoprecipitation using anti‐GFP or anti‐HA antibodies.

### Immunoprecipitation‐Mass Spectrometry (IP‐MS)

To identify HINT3‐interacting proteins, immunoprecipitation followed by mass spectrometry (IP‐MS) was performed. Proteins immunoprecipitated from samples were separated by SDS‐PAGE, and the corresponding gel bands were excised into 1 mm^3^ pieces. Gel pieces were washed with ultrapure water for 10 min, followed by destaining with 50% acetonitrile (ACN) in 100 mM NH_4_HCO_3_ (pH 8.0) until clear. The gel pieces were dehydrated with 100% ACN for 10 min, and residual liquid was removed before vacuum drying. Reduction was carried out by incubating the gel pieces with 10 mM DTT in 50 mM NH_4_HCO_3_ (pH 8.0) at 56 °C for 1 h. After removal of the solution, the gel pieces were dehydrated again with 100% ACN and dried. Alkylation was performed with 60 mM IAA in 50 mM NH_4_HCO_3_ in the dark for 30 min at room temperature. Following alkylation, the gel pieces were washed, dehydrated, and dried as before. Proteins were digested with trypsin in 50 mM NH_4_HCO_3_ at 37 °C overnight. Peptides were extracted by incubating gel pieces with 0.1% formic acid (FA) in ACN for 5 min, followed by brief sonication and centrifugation. This extraction process was repeated three times, and the supernatants were pooled, vacuum‐dried, and stored at ‐20 °C until analysis. Prior to LC‐MS/MS, the peptides were reconstituted in 0.1% FA. Mass spectrometry analysis was performed using a Thermo Scientific Q Exactive mass spectrometer. Raw data were processed using Protein Discoverer (v2.3) with the Sequest HT algorithm. Data were searched against the UniProt mouse proteome database (UniProt_Mouse_202201.fasta) with the following parameters: trypsin as the digestion enzyme, dynamic modifications including oxidation, acetylation, and carbamidomethylation, and filtering thresholds set at PSM Maximum Delta Cn and Maximum Rank ≥ 0.05. Contaminants and reverse hits were excluded, and the remaining identified proteins were used for subsequent analysis.

### Mitochondrial Membrane Potential Assessment (JC‐1 Staining)

Mitochondrial membrane potential (MMP) was evaluated using the JC‐1 assay (Beyotime, China) following the manufacturer's protocol. Cultured cells were seeded onto appropriate culture dishes or multi‐well plates and allowed to reach ≈70‐80% confluency under standard conditions (37 °C, 5% CO_2_). The cells were then incubated with a freshly prepared JC‐1 dye solution at a final concentration of 5 µM for 20 min at 37 °C in the dark to prevent photobleaching. After incubation, excess dye was removed by gently washing the cells three times with cold phosphate‐buffered saline (PBS), allowing 2 min between each wash to ensure thorough removal of unbound dye. Immediately following the washing steps, the cells were imaged using a fluorescence microscope equipped with the appropriate filters for JC‐1 (excitation ≈488 nm; emission at ≈530 nm for green fluorescence and 590 nm for red fluorescence). Consistent imaging parameters, such as exposure time and gain, were maintained across all samples to ensure reliable comparisons. The red‐to‐green fluorescence intensity ratio, indicative of MMP, was quantified using ImageJ software.

### siRNA Knockdown and Adenoviral Overexpression

To knockdown HINT3 expression, AC16 cells were transfected with HINT3‐specific siRNA (HANBIO, China) using Lipo6000 Transfection Reagent (Beyotime, China) according to the manufacturer's instructions. Negative control siRNA was used as a control. For overexpression studies, adenoviral vectors encoding HINT3 (HANBIO, China) were constructed and transduced into AC16 cells at a multiplicity of infection (MOI) of 100.

### TUNEL Staining

Apoptosis in cardiac tissues was assessed using TUNEL (Terminal deoxynucleotidyl transferase dUTP nick end labeling) staining. Heart sections were fixed in 4% paraformaldehyde, permeabilized with 0.1% Triton X‐100, and incubated with TUNEL reaction mixture according to the manufacturer's protocol (Beyotime, China). Nuclei were counterstained with DAPI. Apoptotic cells were quantified using fluorescence microscopy and ImageJ software.

### Succinate Dehydrogenase (SDH) Activity Assay

The activity of succinate dehydrogenase (SDH) was determined by measuring its ability to catalyze the oxidation of succinate to fumarate. In this reaction, the hydrogen released from succinate oxidation was transferred via phenazine methosulfate (PMS) to reduce 2,6‐dichlorophenolindophenol (DCPIP), a redox dye with a characteristic absorbance peak at 600 nm. By monitoring the decrease in absorbance at 600 nm using a spectrophotometer, the rate of DCPIP reduction was quantified, which directly reflects the enzymatic activity of SDH (mlbio, China).

### Statistical Analysis

All statistical analyses were performed using GraphPad Prism software (10.1.2). Data were presented as mean ± standard deviation (SD). For comparisons between two independent groups, unpaired two‐tailed Student's t‐test was applied. For experiments involving more than two groups with a single independent variable, one‐way analysis of variance (one‐way ANOVA) followed by Tukey's post hoc test was used. When two independent variables (e.g., genotype and treatment) were involved, two‐way ANOVA with appropriate post hoc testing was performed to analyze main effects and interactions. A p‐value less than 0.05 was considered statistically significant.

## Conflict of Interest

The authors declare no conflict of interest.

## Author Contributions

J.Y., Q.Y., and T.H. are co–first authors and contributed equally to this work. J.Y. and Q.T. conceived and designed the experiments. T.H., Q.Y., Y.Z., and Y.L. performed the experiments. J.Y., Y.X., and Q.‐Q.W. analyzed the data. J.Y. wrote and revised the manuscript.

## Supporting information



Supporting Information

Supporting Information

## Data Availability

The data that support the findings of this study are available from the corresponding author upon reasonable request.
