# Supplementary material for: The Role of HINT3 in Myocardial Ischemia‐Reperfusion Injury in Male Mice: Mechanisms Involving SDHA and its Acetylation
Source: Adv Sci (Weinh). 2025 Aug 4;12(33):e03109. doi: 10.1002/advs.202503109 (PMC12412524; doi:10.1002/advs.202503109)
Supplement: Supplementary file 1 — Supporting Information [file ADVS-12-e03109-s002.docx]

**Supplementary Figure 1**


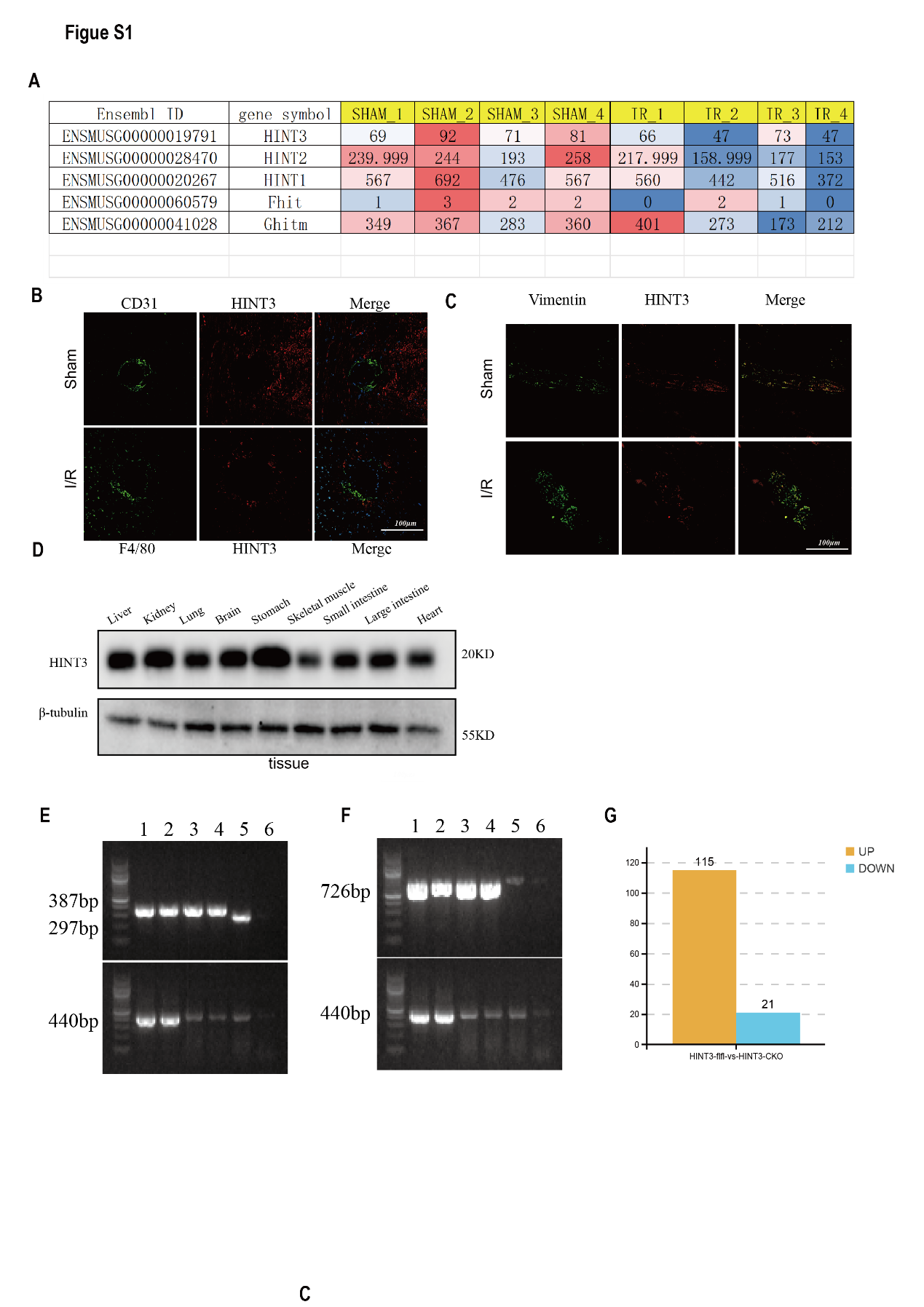


**Supplementary Figure 1**

(A) Heatmap analysis of RNA-sequencing data (GSE255933) from mouse heart tissue comparing SHAM and I/R groups.

(B–C) Immunofluorescence staining of cardiac sections from sham and I/R mice showing HINT3 expression (red) in CD31⁺ endothelial cells (B), Vimentin⁺ fibroblasts (C).

(D) Western blot analysis of HINT3 expression across various mouse tissues.

(E) Representative PCR results for genotyping HINT3^fl/fl^ and HINT3^CKO^ mice. The upper panel shows the presence of a 387 bp band indicating the HINT3^fl/fl^ allele. The lower panel shows a 440 bp band indicating Cre recombinase expression in HINT3^CKO^ mice. Lanes 1 and 2 represent HINT3^CKO^ mice, lanes 3 and 4 represent HINT3^fl/fl^ mice, lane 5 represents wild-type (WT) control mice, and lane 6 is a negative control (no template). This figure confirms the genotypes of HINT3^fl/fl^, HINT3^CKO^, and WT mice using PCR-based analysis.

(F) Representative PCR results for genotyping HINT3 transgenic (Tg) and Cre-positive mice. The upper panel shows a 726 bp band indicating the presence of the HINT3 overexpression transgene. The lower panel shows a 440 bp band indicating Cre recombinase expression in Cre-positive mice. Lanes 1 and 2 represent HINT3 Tg and Cre-positive mice, lanes 3 and 4 represent HINT3 Tg mice without Cre expression, lane 5 represents wild-type (WT) control mice, and lane 6 is a negative control (no template). This figure confirms the genotypes of HINT3 transgenic and Cre-positive mice using PCR-based analysis.

(G) Bar chart showing the number of differentially expressed genes (DEGs) between HINT3^fl/fl^ and HINT3^CKO^ mice. A total of 115 genes were upregulated (orange), and 21 genes were downregulated (blue) in HINT3^CKO^ mice compared to HINT3^fl/fl^ mice.

**Supplementary Figure 2**


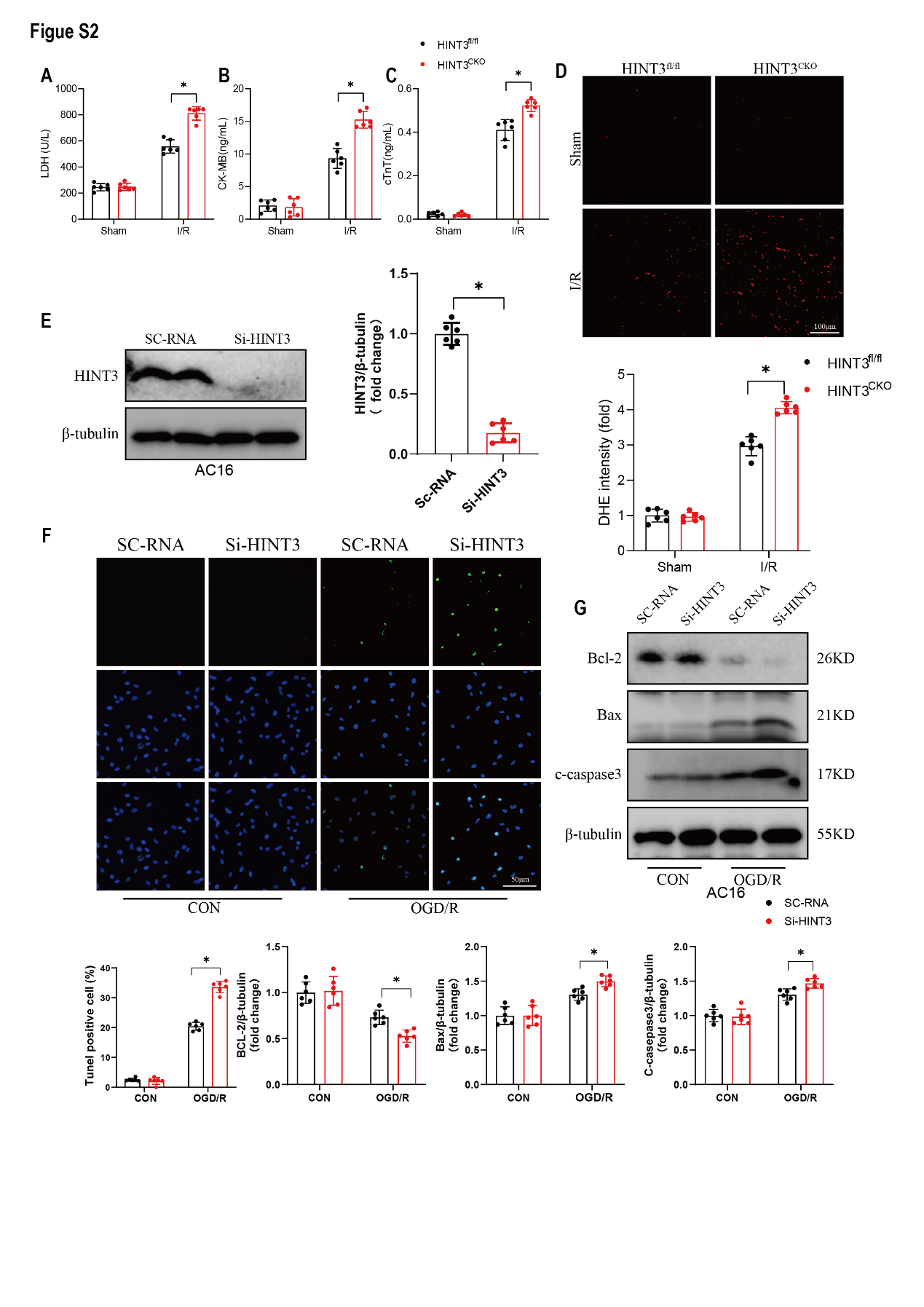


**Supplementary Figure 2**

(A–C) Quantification of serum lactate dehydrogenase (LDH), creatine kinase-MB (CK-MB), and cardiac troponin T (cTnT) levels from HINT3^fl/fl^ and HINT3^CKO^ mice after I/R injury (n = 6).

(D) Representative DHE staining and quantification of ROS levels in cardiac tissue from HINT3^fl/fl^ and HINT3^CKO^ mice after I/R (n = 6). Scale bar: 100 μm.

(E) Western blot and quantification confirming siRNA-mediated knockdown of HINT3 in AC16 cells under OGD/R (n = 6).

(F) Representative TUNEL staining and quantification of apoptotic nuclei in AC16 cells treated with si-HINT3 or scrambled RNA under normoxic or OGD/R conditions (n = 6). Scale bar: 50 µm.

(G) Western blot analysis and quantification of apoptosis markers (BCL-2, BAX, cleaved-caspase3) in AC16 cells with or without HINT3 knockdown under OGD/R (n = 6).

**Supplementary Figure 3**


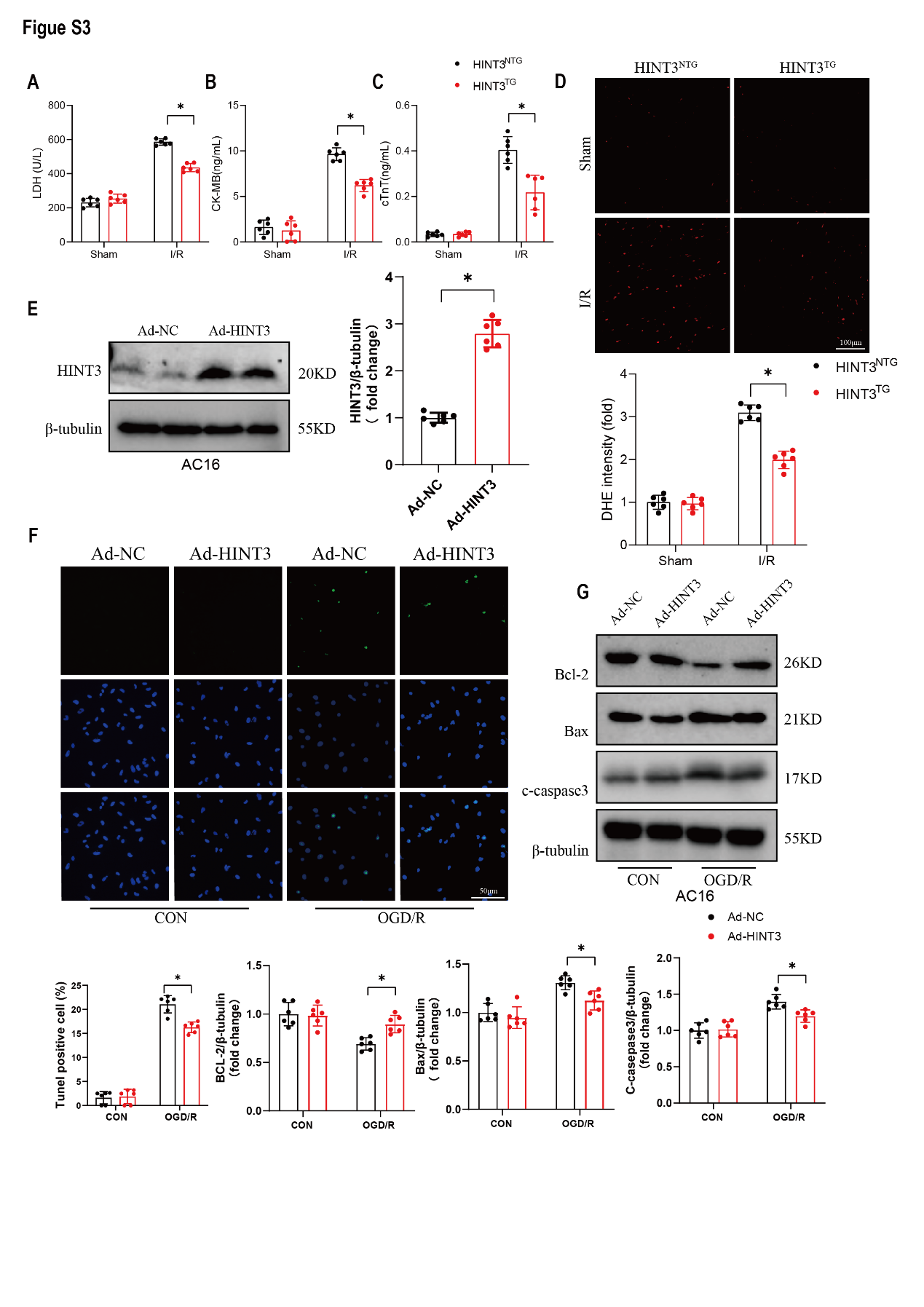


**Supplementary Figure 3**

(A–C) Serum levels of LDH, CK-MB, and cTnT in HINT3^TG^ and HINT3^NTG^ mice subjected to sham or I/R surgery (n = 6).

(D) Representative DHE staining of cardiac tissue showing ROS levels in HINT3^TG^ and HINT3^NTG^ mice under sham or I/R conditions. Red fluorescence indicates superoxide production (n = 6).

(E) Western blot and quantification of HINT3 expression in AC16 cells transfected with control or HINT3-overexpressing adenovirus (n = 6).

(F) Representative TUNEL and DAPI staining of AC16 cells infected with Ad-HINT3 or Ad-NC and exposed to OGD/R. Quantification of TUNEL-positive cells is shown (n = 6). Scale bar: 50 µm.

(G) Western blot analysis of Bcl-2, Bax, and cleaved caspase-3 in AC16 cells following OGD/R with or without HINT3 overexpression (n = 6).

**Supplementary Figure 4**


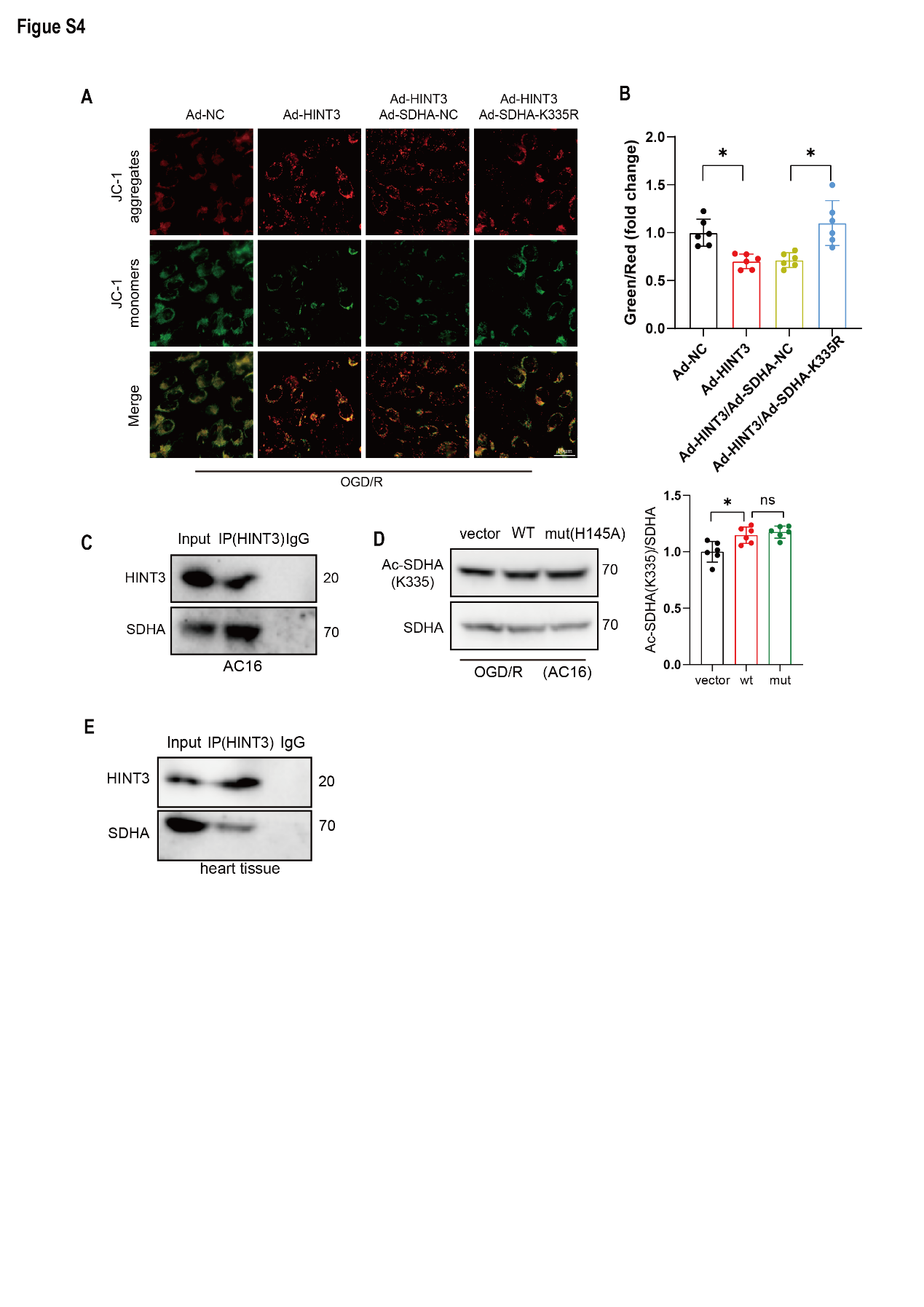


**Supplementary Figure 4**

(A) JC-1 staining for mitochondrial membrane potential in AC16 cells co-treated with Ad-HINT3 and Ad-SDHA-K335R or controls under OGD/R.

(B) Quantification of green/red fluorescence ratio from (A), indicating mitochondrial membrane potential (n = 6).

(C) Co-IP in AC16 cells showing HINT3 (WT or H145A mutant) binds SDHA.

(D) Western blot showing SDHA-K335 acetylation in AC16 cells expressing HINT3 WT or H145A mutant under OGD/R (n = 6).

(E) Co-immunoprecipitation (Co-IP) analysis using mitochondrial protein fractions isolated from mouse heart tissue.

**Supplementary Figure 5**


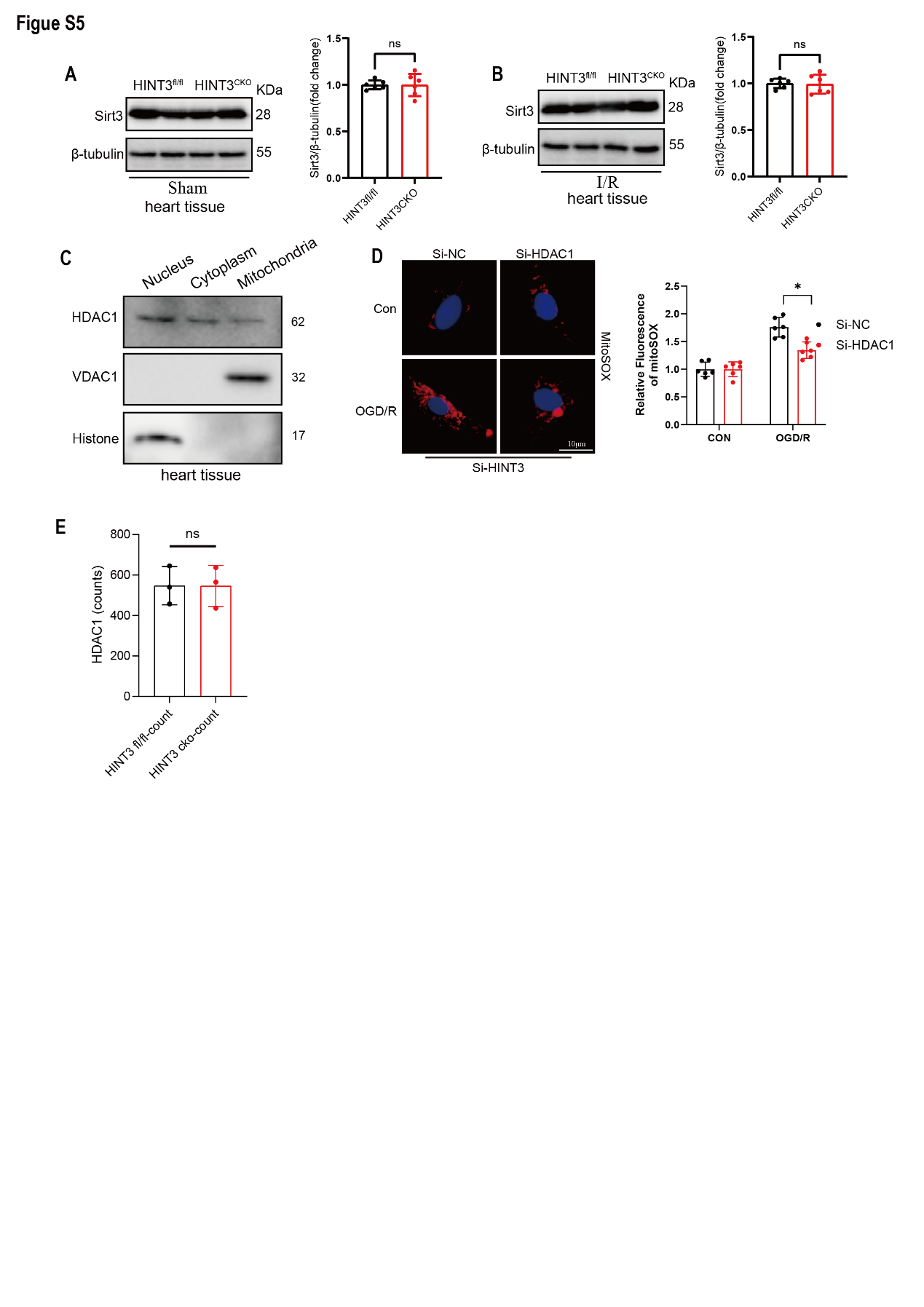


**Supplementary Figure 5**

(A–B) Western blot showing SIRT3 levels in HINT3^fl/fl^ and HINT3^CKO^ mice under sham and I/R (n = 6).

(C) Subcellular localization of HDAC1 in nuclear, cytoplasmic, and mitochondrial fractions from heart tissue. VDAC1 and Histone used as markers.

(D) MitoSOX staining in AC16 cells showing HDAC1 knockdown reverses HINT3 knockdown-induced mitochondrial ROS under OGD/R (n = 6). Scale bar: 10 µm.

(E) HDAC1 transcript levels from RNA-seq data in HINT3^fl/fl^ vs. HINT3^CKO^ hearts after I/R (n = 3). No significant change observed.

**Supplementary Figure 6**


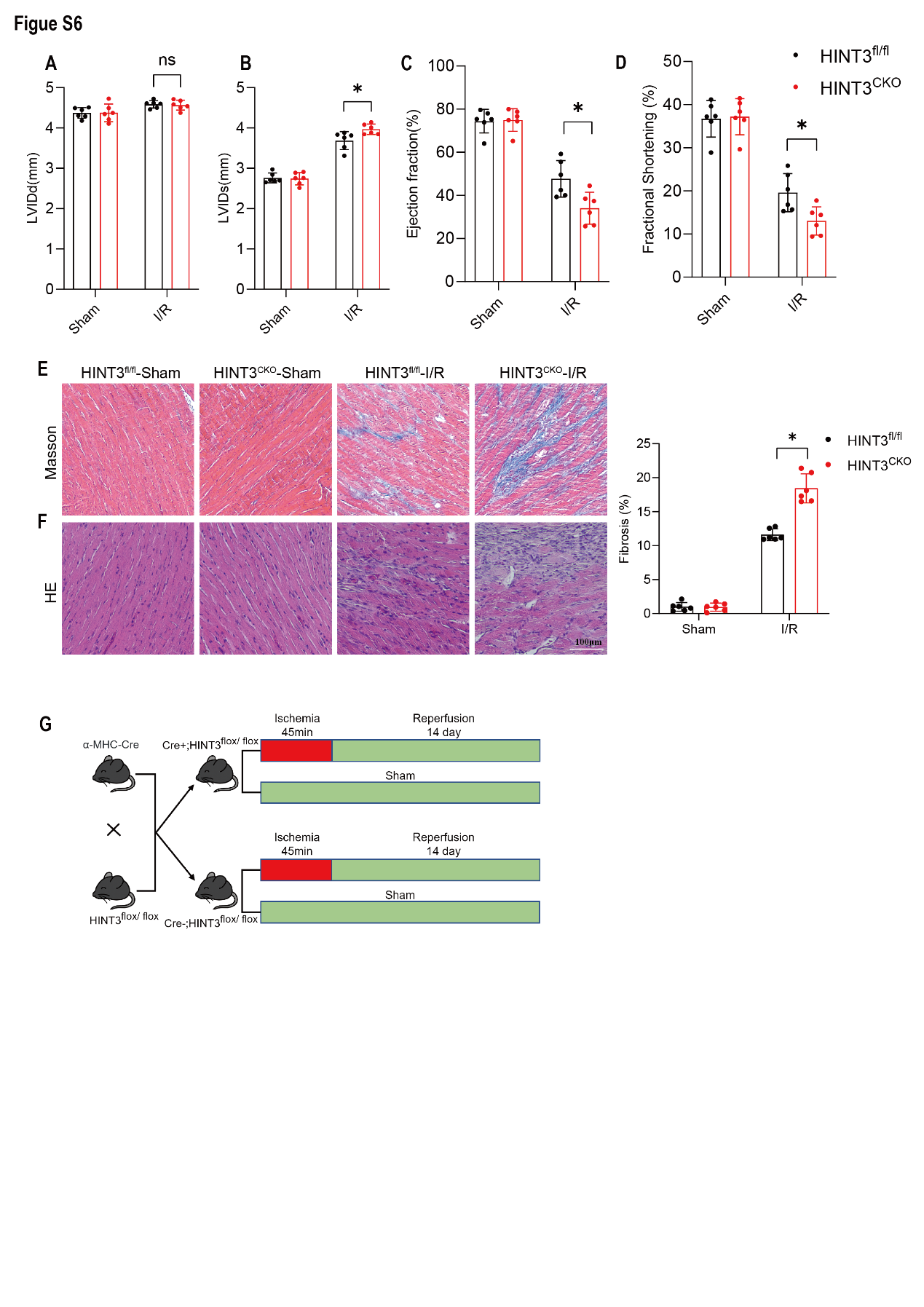


**Supplementary Figure 6**

(A-D) Echocardiographic assessment of left ventricular end-diastolic diameter (LVIDd), end-systolic diameter (LVIDs), fractional shortening (FS), and ejection fraction (EF) in sham and I/R groups (n = 6).

(E) Representative images of Masson's trichrome staining showing myocardial fibrosis (blue area) in heart sections from each group (scale bar = 100 μm).

(F) Representative images of hematoxylin-eosin (HE) staining showing myocardial morphology (scale bar = 100 μm).

(G) Diagram showing the experimental protocol for myocardial I/R injury (45 min ischemia followed by 14 day reperfusion).
